# Impaired interferon response in senecavirus A infection and identification of 3C^pro^ as an antagonist

**DOI:** 10.1128/jvi.00585-24

**Published:** 2024-06-13

**Authors:** Xiangle Zhang, Pengfei Li, Wenzhe Chen, Shilei Zhang, Kangli Li, Yi Ru, Zhenxiang Zhao, Weijun Cao, Fan Yang, Hong Tian, Jianhong Guo, Jijun He, Zixiang Zhu, Haixue Zheng

**Affiliations:** 1 State Key Laboratory of Veterinary Etiological Biology, College of Veterinary Medicine, Lanzhou University, Lanzhou Veterinary Research Institute, Chinese Academy of Agricultural Sciences, Lanzhou, China; University of North Carolina at Chapel Hill, Chapel Hill, North Carolina, USA

**Keywords:** senecavirus A, 3C protease, interferon response

## Abstract

Senecavirus A (SVA), a picornavirus, causes vesicular diseases and epidemic transient neonatal losses in swine, resulting in a multifaceted economic impact on the swine industry. SVA counteracts host antiviral response through multiple strategies facilitatng viral infection and transmission. However, the mechanism of how SVA modulates interferon (IFN) response remains elusive. Here, we demonstrate that SVA 3C protease (3C^pro^) blocks the transduction of Janus kinase-signal transducer and activator of transcription (JAK-STAT) signaling pathway to antagonize type I IFN response. Mechanistically, 3C^pro^ selectively cleaves and degrades STAT1 and STAT2 while does not target JAK1, JAK2, and IRF9, through its protease activity. Notably, SVA 3C^pro^ cleaves human and porcine STAT1 on a Leucine (L)-Aspartic acid (D) motif, specifically L693/D694. In the case of STAT2, two cleavage sites were identified: glutamine (Q) 707 was identified in both human and porcine, while the second cleavage pattern differed, with residues 754–757 (Valine-Leucine-Glutamine-Serine motifs) in human STAT2 and Q758 in porcine STAT2. These cleavage patterns by SVA 3C^pro^ partially differ from previously reported classical motifs recognized by other picornaviral 3C^pro^, highlighting the distinct characteristics of SVA 3C^pro^. Together, these results reveal a mechanism by which SVA 3C^pro^ antagonizes IFN-induced antiviral response but also expands our knowledge about the substrate recognition patterns for picornaviral 3C^pro^.

**IMPORTANCE:**

Senecavirus A (SVA), the only member in the *Senecavirus* genus within the *Picornaviridae* family, causes vesicular diseases in pigs that are clinically indistinguishable from foot-and-mouth disease (FMD), a highly contagious viral disease listed by the World Organization for Animal Health (WOAH). Interferon (IFN)-mediated antiviral response plays a pivotal role in restricting and controlling viral infection. Picornaviruses evolved numerous strategies to antagonize host antiviral response. However, how SVA modulates the JAK-STAT signaling pathway, influencing the type I IFN response, remains elusive. Here, we identify that 3C^pro^, a protease of SVA, functions as an antagonist for the IFN response. 3C^pro^ utilizes its protease activity to cleave STAT1 and STAT2, thereby diminishing the host IFN response to promote SVA infection. Our findings underscore the significance of 3C^pro^ as a key virulence factor in the antagonism of the type I signaling pathway during SVA infection.

## INTRODUCTION

SVA, a newly emerging picornavirus, causes a vesicular disease in pigs that is clinically indistinguishable from foot-and-mouth disease; this poses a significant productive and economical threat to the pork industry (1–3). SVA is the only member of *Senecavirus* genus within the *Picornaviridae* family (4). It contains a single-stranded, 7.2 kb positive-sense RNA genome that encodes a large precursor polyprotein. This viral polyprotein is processed into four mature structural proteins (VP1, VP2, VP3, and VP4) and eight mature non-structural proteins (L, 2A, 2B, 2C, 3A, 3B, 3C, and 3D) by viral proteases including 3C^pro^ (4). The protease activity of SVA 3C^pro^ is based on its catalytic triad that comprises three conserved residues, His48, Asp84, and Cys160 (4). These catalytic residues are vital for the protease activity of SVA 3C^pro^, with the loss of either one being able to disrupt viral replication. Other than the essential role in viral replication, SVA 3C^pro^ has been reported to dysregulate multiple host cellular signaling pathways, such as interferon production, pyroptosis, and apoptosis (5–8).

Viral infection triggers cellular pattern recognition receptors (PRR) regulated-signaling pathways to produce cytokines and chemokines among which type I interferon (IFN) plays a vital role in establishing the antiviral response (9, 10). Secreted IFN binds to specific cell surface dimeric receptors, named IFN-α/β receptor (IFNAR) (9). This binding initiates a signaling cascade, referred to as Janus kinase-signal transducers and activators of transcription (JAK-STAT) pathways (11). During this process, STAT1 and STAT2 are phosphorylated by activated Janus kinase 1 (JAK1) and tyrosine kinase 2 (TYK2). Subsequently, they interact with interferon regulatory 9 (IRF9) to form a transcriptional complex called interferon-stimulated gene factor 3 (ISGF3). ISGF3 translocates into the nucleus, where it binds to interferon-stimulated response elements, leading to the expression of hundreds of IFN-stimulated genes (ISGs) (9, 12); these ISGs exert diverse antiviral effector functions to suppress virus infection (13, 14).

Given the crucial role of the JAK-STAT pathway in regulating antiviral response, viruses evolve various strategies to disrupt its functions (15, 16), including picornavirus. Previous studies have shown that FMDV, EV71, and PKV utilize diverse mechanisms to negatively modulate the IFN-induced JAK-STAT pathway transduction (17–20). Unlike these mechanisms, our study found that SVA 3C^pro^ targeted STAT1 and STAT2 simultaneously, cleaving and degrading these two critical transcription factors to suppress antiviral genes response induced by type I IFN. Further investigation showed that the protease activity of SVA 3C^pro^ was responsible for the cleavage and degradation of STAT1 and STAT2. We also demonstrated that SVA 3C^pro^ recognizes non-classical residues on STAT1 and STAT2 to mediate the cleavage. These findings highlight a novel mechanism through which SVA 3C^pro^ antagonizes the IFN-regulated antiviral signaling pathway.

## RESULTS

### SVA infection diminishes IFN-induced antiviral response

**Fig 1:**
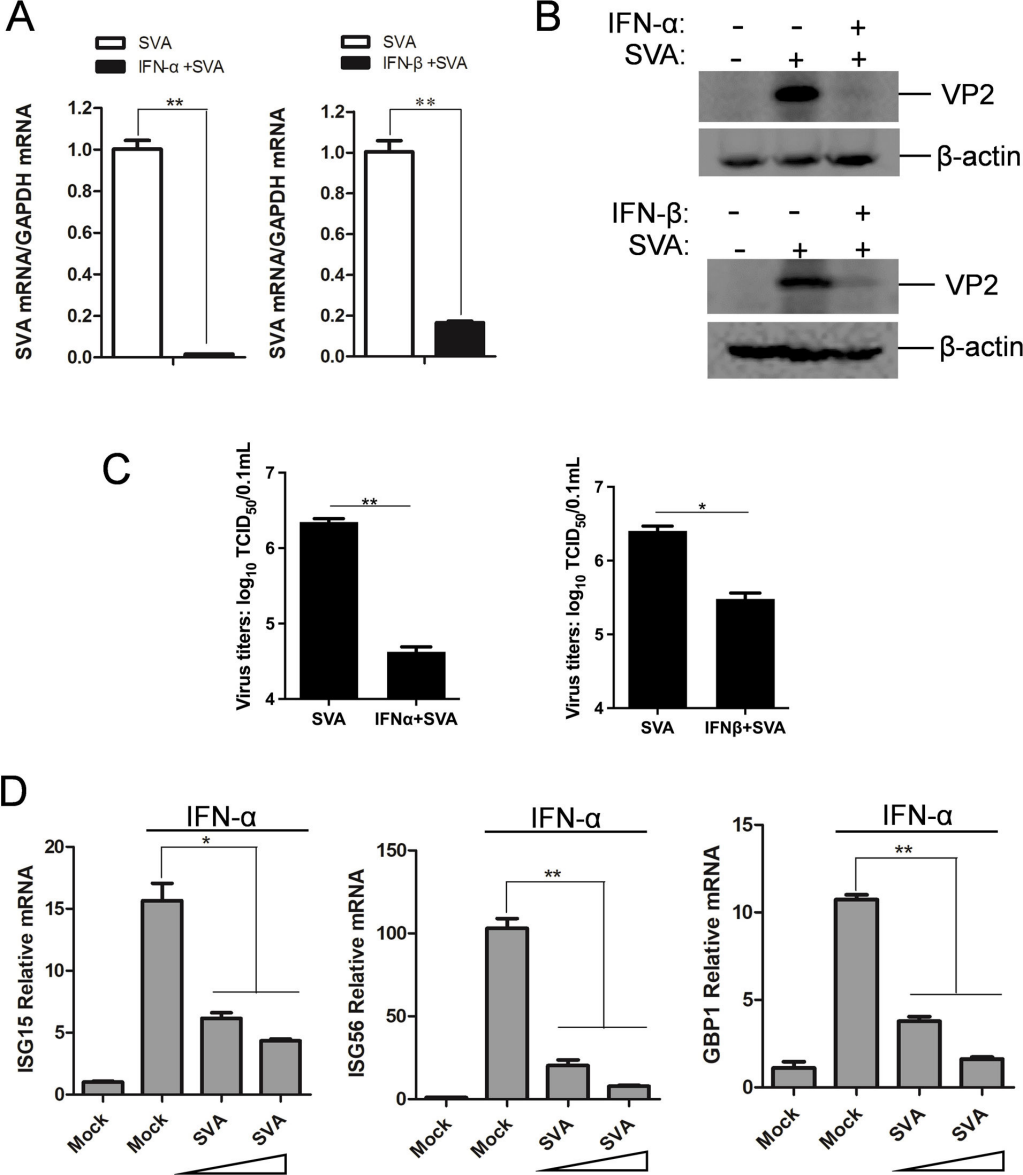
SVA antagonizes type I IFN-induced antiviral response. (**A–C**) HEK-293T cells treated with or without IFN-α (1,000 U/mL) or IFN-β (1,000 U/mL) (1,000 U/mL) for 12 h were infected with SVA infection (0.1 MOI). At 12 h post-infection, cell lysates were collected to assess the levels of viral mRNA (**A**) and protein (VP2) (**B**), and supernatants were harvested to measure viral titer (**C**). (**D**) HEK-293T cells infected with 0.05 or 0.1 MOI of SVA or without infection for 6 h were treated with IFN-α for 6 h. mRNA levels of *ISG15*, *ISG56,* and *GBP1* were measured by qPCR. All experiments were repeated at least three times. *P < 0.05, **P < 0.01.

Previous studies have shown that SVA infection antagonizes IFN production (5, 21, 22), whereas how SVA modulates IFN-induced antiviral response has not been investigated. We first examined the sensitivity of SVA infection to type I IFN treatment. To do this, HEK-293T cells were treated with IFN-α or IFN-β for 12 h, prior to infection with SVA at 0.1 multiplicity of infection (MOI). Both IFN-α and IFN-β treatment caused substantial reductions in viral mRNA, protein, and viral titer (Fig. 1A through C), indicating SVA is sensitive to IFN-induced antiviral response. This finding implied that SVA may have evolved strategies to counteract IFN response, potentially facilitating its infection. To address this question, we evaluated whether SVA infection had the ability to suppress IFN-induced antiviral response. Type I IFN activates JAK-STAT signaling pathway, leading to the upregulated expression of ISGs, which plays a critical role in restricting virus replication. HEK-293T cells were infected with SVA at 0.05 or 0.1 MOI (The single replication cycle of SVA in HEK-293T cells is approximately 3 hours, as shown in Fig. S2E.) and after 6 h or 12 h were treated with IFN-α for additional 6 h. mRNA levels of ISGs including ISG15, ISG56, ISG54, MxA, and GBP1 in SVA-infected cells were significantly lower than those in uninfected cells (Fig. 1D; Fig. S1G). These data suggest that SVA is sensitive to IFN treatment and it might has the ability to diminish IFN-induced antiviral response.

### SVA 3C^pro^ suppresses type I IFN-induced antiviral responses

**Fig 2:**
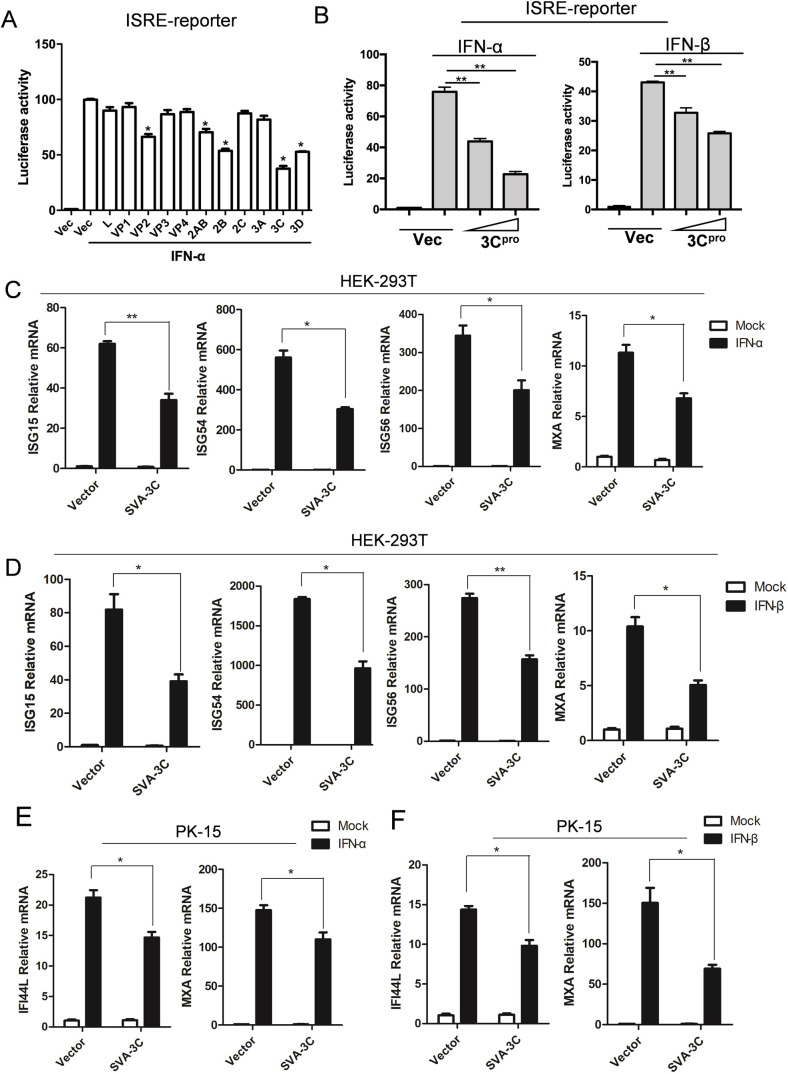
SVA 3C^pro^ suppresses type I IFN-induced antiviral responses. (**A**) HEK-293T cells were transfected with ISRE reporter plasmids (50 ng), internal control plasmid PRL-TK (5 ng), and empty vector (Vec) or the indicated viral protein-expressing plasmids (50 ng) for 24 h, transfected-cells were treated with IFN-α (1000 U/mL) or PBS for another 12 h. Cell lysates were harvested for luciferase activity measured by dual luciferase assay. (**B**) HEK-293T cells were transfected with increasing doses of plasmids expressing 3C^pro^ (0, 25, or 50 ng), together with ISRE reporter plasmids and PRL-TK plasmids for 24 h, followed by IFN-α-treatment (left) or IFN-β-treatment (right) for an additional 12 h. The luciferase activity was determined by dual luciferase assay. (**C and D**) HEK-293T cells were transfected with empty vector or 3C^pro^ expressing plasmids for 24 h, followed by IFN-α- (**C**) or IFN-β-treatment (**D**) for 12 h. mRNA levels of the indicated ISGs (*ISG15*, *ISG54*, *ISG56,* and *MxA*) were measured by qPCR. (**E and F**) PK-15 cells were transfected with empty vector or 3C^pro^ expressing plasmids for 24 h, followed by IFN-α- (**E**) or IFN-β (**F**) -treatment for 12 h. Levels of the mRNA of indicated ISGs (*IFI44L* and *MXA*) were determined by qPCR. All experiments were repeated three times. *P < 0.05, **P < 0.01 (The expression of SVA viral proteins in luciferase and RT-qPCR assay were detected by western blot, as shown in Fig. S1).

Picornaviruses utilize their viral proteins to modulate the host antiviral signaling pathway for benefiting infection and transmission (16). To test the effect of SVA viral proteins on type I IFN induced the transduction of JAK-STAT signaling pathway, we transfected HEK-293T cells with plasmids encoding individual SVA viral protein or vector, along with ISRE-luciferase and pRL-TK reporter plasmids for 24 h, followed by stimulation with IFN-α treatment for additional 12 h. The overexpression of VP2, 2AB, 2B, 3C^pro^, and 3D^pol^ significantly suppressed IFN-α-induced ISRE promoter activity (Fig. 2A), with 3C^pro^ displaying the most pronounced inhibitory activity. Moreover, SVA 3C^pro^ overexpression resulted in a sharply reduction of ISRE-luciferase activity induced by IFN-α or IFN-β in a dose-dependent manner (Fig. 2B). We therefore chose 3C^pro^ for further investigation. To verify the inhibited activity of 3C^pro^ on IFN response, we then examined the levels of endogenous ISG transcripts in the context of 3C^pro^ overexpression. HEK-293T cells were transfected with empty vector or plasmids expressing 3C^pro^ for 24 h and then were treated with IFN-α or IFN-β for 12 h. mRNA levels of ISG15, ISG54, ISG56, and MxA induced by IFN-α (Fig. 2C) or IFN-β (Fig. 2D) were significantly reduced when overexpressed 3C^pro^. Similar results were observed in PK-15 cells, another cell line susceptible to SVA infection. Upon stimulating with IFN-α or IFN-β, the mRNA levels of ISGs (IFI44L and MXA) in cells expressing 3C^pro^ were lower compared to those in vector-transfected cells (Fig. 2E and F). The relative protein expression of the above experiments is shown in Fig. S1A through F. Collectively, these data demonstrated SVA 3C^pro^ function as an antagonist for type I IFN-induced antiviral responses.

**Fig 3:**
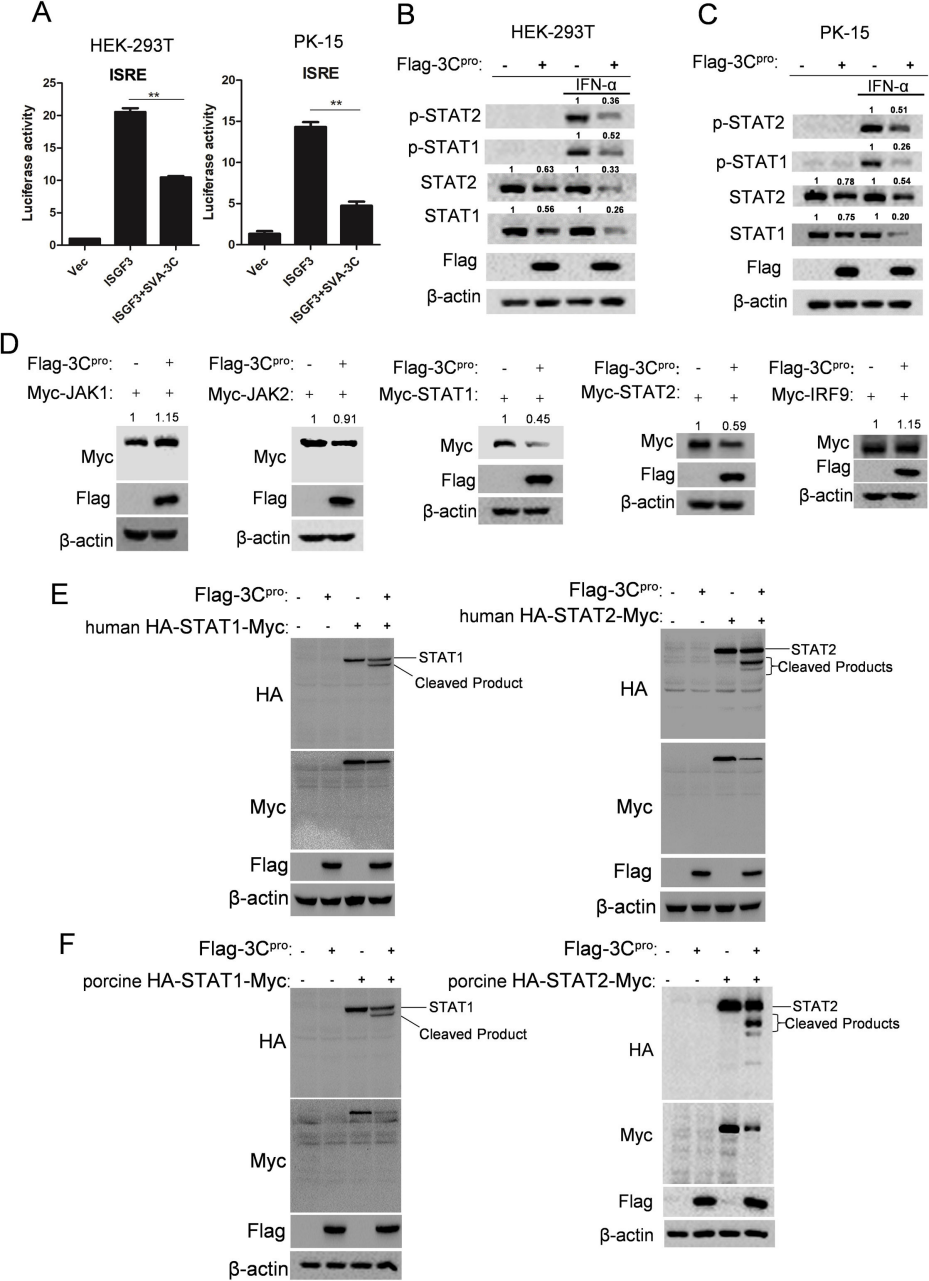
SVA 3C^pro^ disrupts STATs function through induction of the cleavage of both STAT1 and STAT2. (**A**) HEK-293T cells (left) or PK-15 cells (right) were transfected with vector or 3C^pro^ expressing plasmids, and empty vector or STAT1, STAT2, and IRF9 expressing plasmids, together with ISRE reporter plasmids and PRL-TK plasmids for 24 h, the luciferase activity was measured by dual luciferase assay. *P < 0.05, **P < 0.01. (**B and C**) HEK-293T cells (**B**) or PK-15 cells (**C**) were transfected with empty vector or 3C^pro^ expressing plasmids for 12 h, followed by treatment with PBS or IFN-α for 1 h. Levels of STAT1, STAT2, and phosphorylated STAT1 and STAT2 were determined by Western blotting. (**D**) HEK-293T cells were co-transfected with JAK1, JAK2, STAT1, STAT2, or IRF9 expressed plasmids (human) and empty vector or 3C^pro^ plasmids for 24 h, the levels of the indicated proteins were determined by Western blotting using appropriate antibodies. (**E**) HEK-293T cells were co-transfected with the plasmid-expressing human HA-STAT1-myc (left) or human HA-STAT2-myc (right) and empty vector or 3C^pro^ plasmids for 24 h. Protein levels of the indicated molecules were measured by western blotting using appropriate antibodies. (**F**) A similar process to that in (**E**), but using porcine HA-STAT1-myc plasmid (left) or porcine HA-STAT2-myc plasmid (right) instead. All experiments were repeated three times. In B, C, and D, the intensity of indicated protein was normalized to actin and then compared to the Vec group.

Upon IFN stimulation, STAT1, STAT2, and IRF9 form a complex, termed the IFN-stimulated gene factor 3 (ISGF3), which binds to the ISRE promoter and induces the transcription of antiviral genes. We investigated the influence of SVA 3C^pro^ on the ISGF3-regulated ISRE promoter activity. HEK-293T cells were transfected with plasmid-expressing STAT1, STAT2, and IRF9, along with either vector or plasmid-expressing SVA 3C^pro^, in addition to ISRE reporter and PRL-TK plasmids. When assessed luciferase activity at 24 h post-transfection, ISGF3-induced ISRE reporter activity was significantly suppressed in the presence of SVA 3C^pro^ (Fig. 3A, left). Consistently, the overexpression of SVA 3C^pro^ also inhibited ISGF3-induced ISRE reporter activity in PK-15 cells, which are susceptible to SVA infection (Fig. 3A, right). These data are consistent with the above-mentioned findings that SVA 3C^pro^ blocked the type I IFN-induced JAK-STAT pathway transduction. It also implies that SVA 3C^pro^ might target ISGF3 or an unknown molecule downstream of ISGF3.

### SVA 3C^pro^ cleaves STAT1 and STAT2 to antagonize IFN response

To investigate the underlying mechanism by which SVA 3C^pro^ blocks type I IFN-stimulated antiviral response, we examined the impact of 3C^pro^ on the molecules of the JAK-STAT signaling pathway. HEK-293T cells were transfected with 3C^pro^ construct or vector for 12 h, prior to treating with IFN-α. The overexpression of 3C^pro^ resulted in decreased protein levels of endogenous STAT1 and STAT2 in the presence or absence of IFN-α treatment. In addition, 3C^pro^ impaired the phosphorylation of STAT1 and STAT2 following treatment with IFN-α (Fig. 3B). Similar findings were observed in PK-15 cells (Fig. 3C). Previous picornaviral 3C^pro^ has been reported to cleave cellular molecules using its protease activity (5, 17, 23–25). We then examined whether SVA 3C^pro^ played a role in cleaving any molecules within the JAK-STAT signaling pathway. HEK-293T cells were transfected with 3C^pro^ construct, along with the constructs of JAK1, JAK2, STAT1, STAT2, or IRF9. The expression of STAT1 and STAT2 was significantly affected by 3C^pro^, while JAK1, JAK2, and IRF9 were not affected (Fig. 3D), which is consistent with the results mentioned above. As cleaved bands were not observed in the STAT1 and STAT2, we hypothesized that these cleavage sites might be close to the position of myc-tag in the plasmids. Consequently, we added an HA-tag at the N-terminal of STAT1 and STAT2 constructs. Interestingly, cleaved products were observed for both STAT1 and STAT2 in the context of 3C^pro^ overexpression when stanning with HA antibody; one faster-moving band was detected in STAT1 (Fig. 3E, left), and two were found in STAT2 (Fig. 3E, right). Since the amino acid sequence between human and porcine STAT1/2 is highly conserved, we constructed plasmids expressing porcine STAT1 and porcine STAT2 and tested their expression in the presence of SVA 3C^pro^. We observed similar cleaved bands in porcine and human STAT1/2 (Fig. 3F), suggesting similar cleavage sites might be present in them.

**Fig 4:**
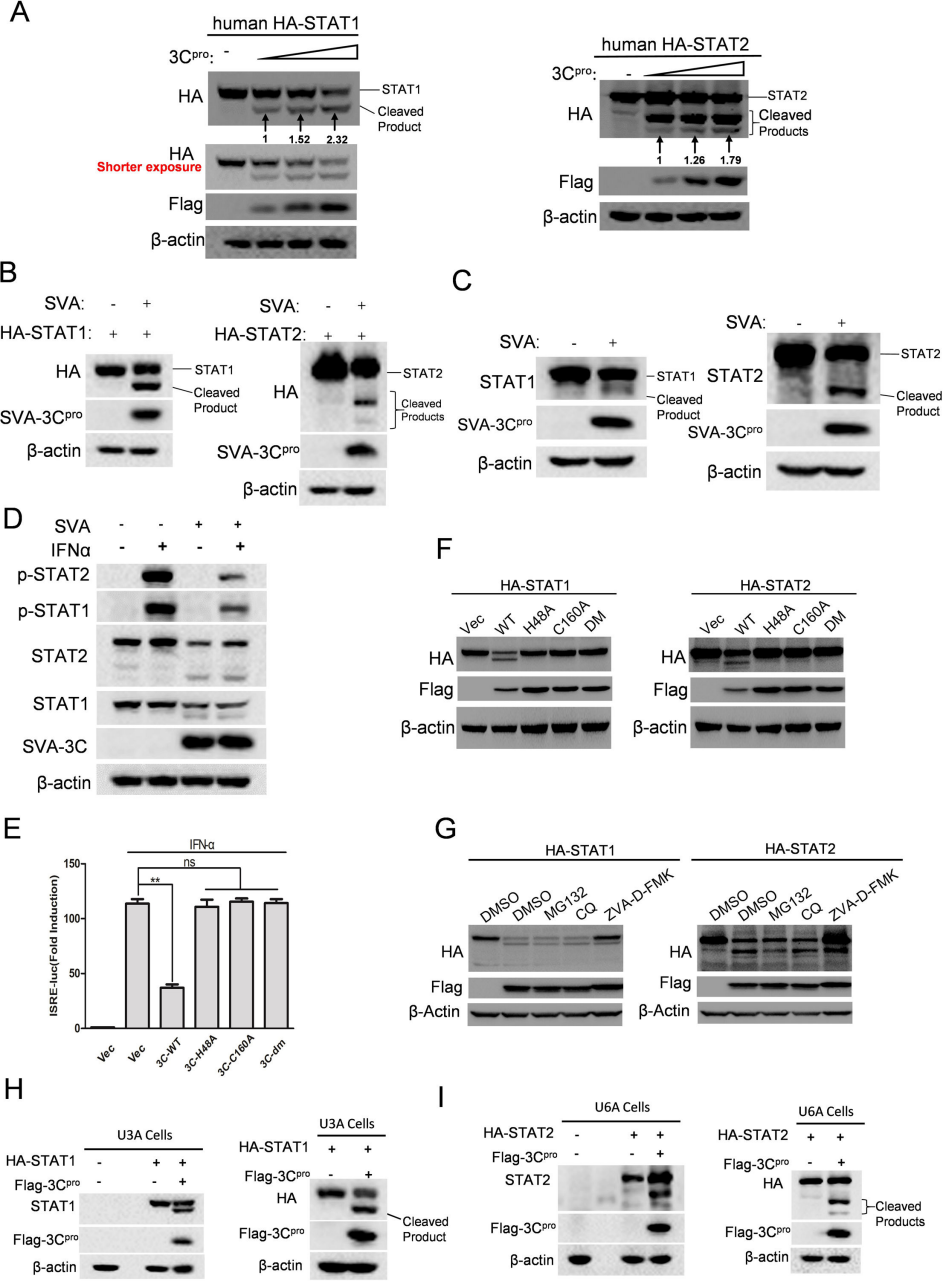
3C^pro^ cleaves and degrades both STAT1 and STAT2 through its protease activity during SVA infection. (**A**) HEK-293T cells were transfected with human-STAT1 (left) or human-STAT2 (right) expressing plasmids, along with increasing amounts of 3C^pro^ expressing plasmids for 24 h. The cells were then lysed and analyzed by Western blotting. The intensity of the cleaved bands of STAT1 and STAT2 (lower molecular weight) were quantified by normalizing them to actin and then comparing them to the lowest dose group. (**B**) HEK-293T cells were transfected with human-STAT1 (left) or human-STAT2 (right) expressing plasmids; after 24 h, the transfected cells were inoculated with or without SVA at MOI = 0.1 for 12 h. Cell lysates were subject to western blotting analysis to measure the expression of indicated proteins. (**C**) HEK-293T cells were mock infected or infected with 0.1 MOI of SVA for 12 h. The expression of endogenous STAT1 or STAT2 proteins was determined by western blotting. (**D**) HEK-293T cells were infected with or without SVA at 0.1 MOI; after 8 h, cells were treated with PBS or IFN-α for 1 h. Cell lysates were collected to analyze protein expression using western blotting with the indicated antibodies. (**E**) HEK-293T cells were transfected with empty vector, 3C^pro^, or 3C^pro^ mutants (H48A, C160A, or H48A/C160A) expressing plasmids, along with ISRE reporter and PRL-TK for 24 h, followed by IFN-α-treatment for 12 h. Luciferase activity was measured by dual luciferase assay. (**F**) HEK-293T cells were transfected with human-STAT1 (left) or human-STAT2 (right) expressing plasmids, along with empty vector, 3C^pro^, or 3C^pro^ mutant plasmid for 24 h. The protein levels of indicated molecules were determined by western blotting. (**G**) HEK-293T cells were transfected human-STAT1 (left) or human-STAT2 (right) expressing plasmid, together with empty vector or 3C^pro^ plasmid. At 6 h post-transfection, cells were maintained in the presence of DMSO, 20 µM of MG132, 50 µM of CQ, or 50 µM of Z-VAD-FMK, as shown in the panel, for an additional 18 h. Western blotting was performed to detect the indicated protein expression. (**H**) U3A (STAT1-null) cells were transfected human-STAT1 expressing plasmids along with empty vector or 3C^pro^ plasmids. The status of STAT1 was detected by anti-STAT1 (left) and anti-HA (right) antibodies using Western blotting. (**I**) Similar to Fig. 4H but U6A (STAT2-null) cells were utilized to assess the cleavage of HA-STAT2 by 3C^pro^. All experiments were repeated three times.

To further validate this finding, we initially evaluated the overexpression of human STAT1/2 in the presence of escalating doses of 3C^pro^. The cleavage of STAT1 and STAT2 gradually intensified as the expression of 3C^pro^ increased (Fig. 4A). Furthermore, we observed progressive cleavage and degradation when STAT1 or STAT2 was co-expressed with SVA 3C^pro^ at various time points (Fig. S2A and B). We also confirmed that other SVA viral proteins, which inhibit ISRE promoter activity, were not able to impact STAT1 and STAT2 (Fig. S2C and D). VP3 and VP1 were selected as controls. Together, these data indicate that 3C^pro^ antagonizes the IFN-induced antiviral response through cleaving STAT1 and STAT2.

We next assess these results in the context of SVA infection. HEK-293T cells were transfected with constructs encoding STAT1 or STAT2 for 24 h and then infected with SVA at 0.1 MOI for 12 h. Consistent with the above-mentioned findings in the 3C^pro^ overexpression context, one faster migrating band for STAT1 (Fig. 4B, left) and two such bands for STAT2 were observed when infected with SVA (Fig. 4B, right). The expressions of endogenous STAT1 and STAT2 were also tested during SVA infection. We noted a reduction in the abundance of STAT1 along with a faintly cleaved band (Fig. 4C, left), as well as a decreased expression of STAT2 with only one cleaved band (Fig. 4C, right). The absence of the other cleaved product in endogenous STAT2 might be due to limitations in antibody recognition. We also analyzed the phosphorylation status of STAT1 and STAT2 in the context of SVA infection. HEK-293T cells were inoculated with or without SVA at 0.1 MOI for 8 h, prior to treatment with IFN-α for 1 h. The phosphorylation levels of STAT1 and STAT2 were reduced in SVA-infected cells compared to those without infection (Fig. 4D), consistent with our previous findings from the overexpression assay in Fig. 3B and C. To rule out any potential influence from the competition of endogenous STAT1 and STAT2 with transfected tagged STAT1 and STAT2, we assessed the cleavage of tagged STAT1 and STAT2 by 3C^pro^ in the U3A (STAT1-null) and U6A (STAT2-null) cells, respectively. The results were consistent with the observation in STAT1 and STAT2 completed cells (Fig. 4H and I). Collectively, these data suggest that SVA 3C^pro^ is responsible for cleaving STAT1 and STAT2 in the virus infection context.

### The protease activity of 3C^pro^ is required for the cleavage and degradation of STAT1 and STAT2

SVA 3C^pro^ possesses a canonical catalytic triad comprised of three conserved residues, His48, Asp84, and Cys160, which are critical for its enzyme activity (4, 26). Previous studies have shown that mutating the His48 or Cys160 residue effectively disrupts the proteolytic activity of SVA 3C^pro^ (5, 6). To further determine the role of protease activity of SVA 3C^pro^ in its antagonistic action on IFN response, we generated three 3C^pro^ mutants, H48A or C160A and a double-site mutation H48A-C160A (DM). We then transfected HEK-293T cells with wild type (WT) 3C^pro^ or either of 3C^pro^ mutants, along with ISRE and pRL-TK luciferase reporter. The result showed that all 3C^pro^ mutants lost the antagonistic effect on ISRE promoter activity (Fig. 4E). To further examine whether the protease activity of 3C^pro^ is implicated in the cleavage of STAT1 and STAT2, HEK-293T cells were transfected with 3C^pro^ or its mutants along with STAT1 or STAT2 for 24 h. Cleavage of both STAT1 (Fig. 4F, left) and STAT2 (Fig. 4F, right) was not observed following transfection with any of 3C^pro^ mutants. We additionally tested 3C^pro^-induced cleavage of STAT1 and STAT2 in the presence of proteasome inhibitor MG132, lysosome inhibitor CQ or caspase inhibitor Z-VAD-FMK. The cleavages of STATs were not affected by any of these inhibitors (Fig. 4G). Notably, in the context of treatment with Z-VAD-FMK, the degradation of STAT1 and STAT2 induced by 3C^pro^ was diminished (Fig. 4G left and right, the fifth lane), suggesting 3C^pro^ may employ another mechanism to degrade STAT1 and STAT2. These results indicate that the protease activity of 3C^pro^ is indispensable in the cleavage of STAT1 and STAT2.

### SVA 3C^pro^ interacts with STAT1 and STAT2 and diminishes the formation and nuclear translocation of ISGF3

**Fig 5:**
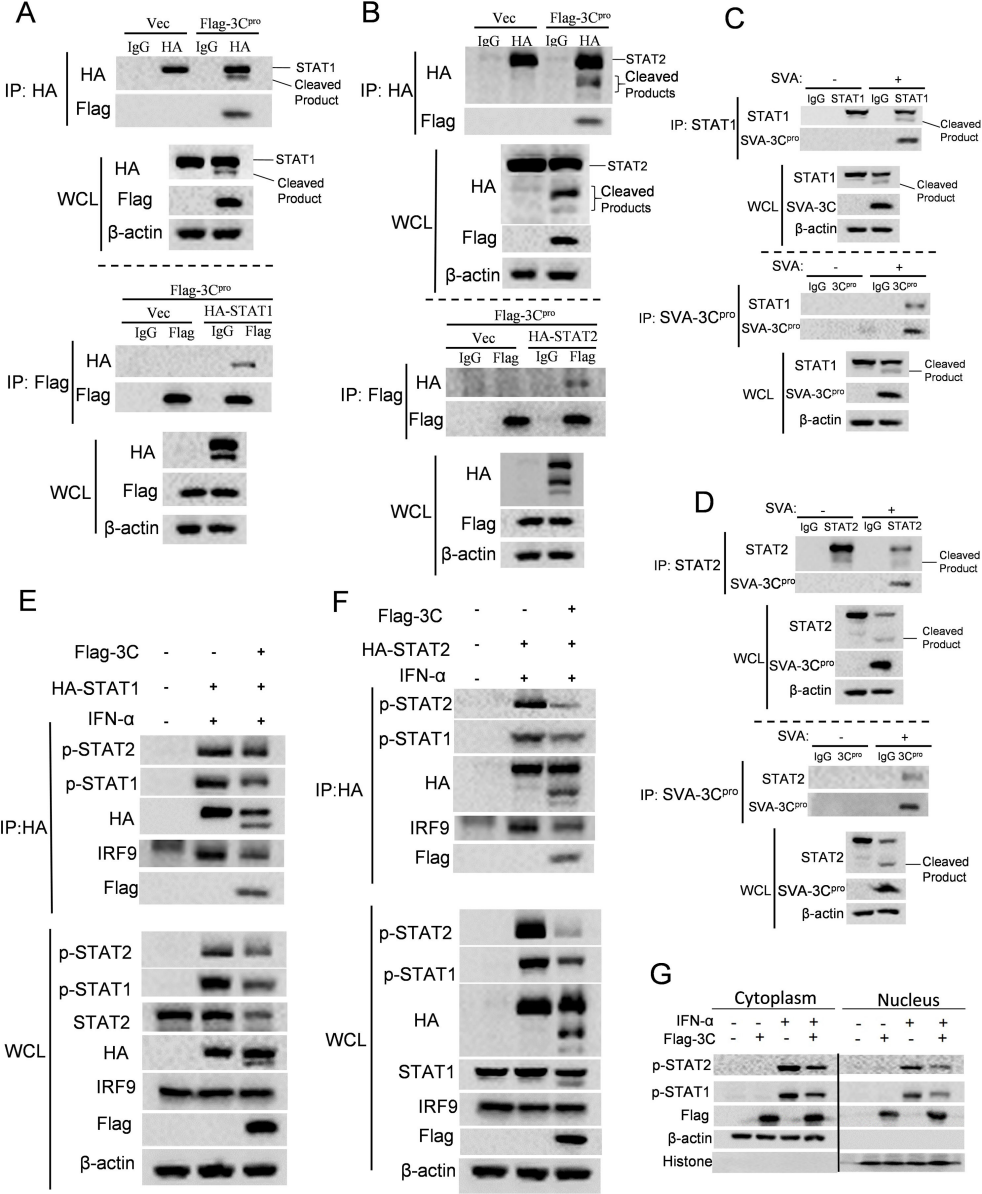
SVA 3C^pro^ interacts with STAT1 and STAT2 and disrupts the formation and nuclear translocation of ISGF3. (**A and B**) HEK-293T cells were transfected with human-STAT1 (**A**) or human-STAT2 (**B**) expressing plasmids, and empty vector or 3C^pro^ expressing plasmids. After 24 h, the cell lysates were collected to perform immunoprecipitated using anti-HA or anti-Flag antibodies. The immunoprecipitated (IP) samples and whole-cell lysates (WCL) sample were subjected to Western blotting analysis using the indicated antibodies. (**C and D**) HEK-293T cells were mock infected or infected with 0.1 MOI of SVA for 12 h. Cell lysates were immunoprecipitated with anti-STAT1 (**C**), anti-STAT2 (**D**), or anti-SVA 3C^pro^ antibodies. IP and WCL were subjected to Western blotting analysis using the indicated antibodies. (**E and F**) HEK-293T cells were transfected with STAT1 (**E**) or STAT2 (**F**) expressing plasmids, and empty vector or 3C^pro^ expressing plasmids for 24 h, then followed by mock treatment or IFN-α treatment for 1 h. The cell lysates were immunoprecipitated with anti-HA antibodies. The immunoprecipitated samples and WCL were subjected to western blotting analysis using the indicated antibodies. (**G**) Empty vector or 3C^pro^ expressing plasmids were transfected into HEK-293T cells for 24 h. After treatment with IFN-α for 1 h, cytoplasmic and nuclear proteins of the cells were collected, as described in the method section. Extracted proteins were analyzed using Western blotting. All experiments were repeated three times.

We sought to investigate whether SVA 3C^pro^-induced STAT1 and STAT2 cleavage is associated with their direct interaction. To do this, HEK-293T cells were transfected with plasmids expressing STAT1 or STAT2 and 3C^pro^, and cell lysate was harvested to perform immunoprecipitation assays. 3C^pro^ was pulled down by either STAT1 (Fig. 5A) or STAT2 (Fig. 5B). Similarly, STAT1 or STAT2 was also pulled down by 3C^pro^. To further determine the interactions in the context of viral infection, HEK-293T cells were inoculated with or without SVA for 12 h and collected for immunoprecipitation assays. Endogenous STAT1 (Fig. 5C) and STAT2 (Fig. 5D) were precipitated with SVA 3C^pro^ in the viral infection context, consistent with the results observed in the overexpression assay. Upon IFN stimulation, IRF9, STAT1, and STAT2 form a complex, ISGF3, which translocates into the nucleus and initiates ISGs expression. To investigate whether 3C^pro^-mediated cleavage of STATs impacts the formation of ISGF3, we transfected HEK-293T cells with plasmids expressing STAT1 or STAT2, with or without 3C^pro^. At 24 h post-transfection, cells were treated with IFN-α for 1 h. The result of immunoprecipitation assay showed the presence of 3C^pro^ reduced the formation of IFN-induced ISGF3 (Fig. 5E and F). We then investigated the nuclear translocation of ISGF3 by extracting nuclear and cytoplasmic proteins and using p-STAT1(Y701) and p-STAT2 (Y690) as the markers. HEK-293 cells were transfected with vector or 3C^pro^ construct for 24 h and cytoplasmic and nuclear proteins were collected after treatment with IFN-α for 1 h. As expected, IFN-α stimulation induced the translocation of p-STAT1 and p-STAT2 into the nucleus (Fig. 5G). In addition, compared to the vector-transfected group, less amount of p-STAT1 and p-STAT2 were observed in the nucleus in the presence of 3C^pro^ (Fig. 5G). These results collectively suggest that 3C^pro^ directly interacts with STAT1 and STAT2, which decreases the ISGF3 formation and nuclear translocation induced by IFN.

### Identification of the cleavage sites in human and porcine STAT1 and STAT2 recognized by SVA 3C^pro^

**Fig 6:**
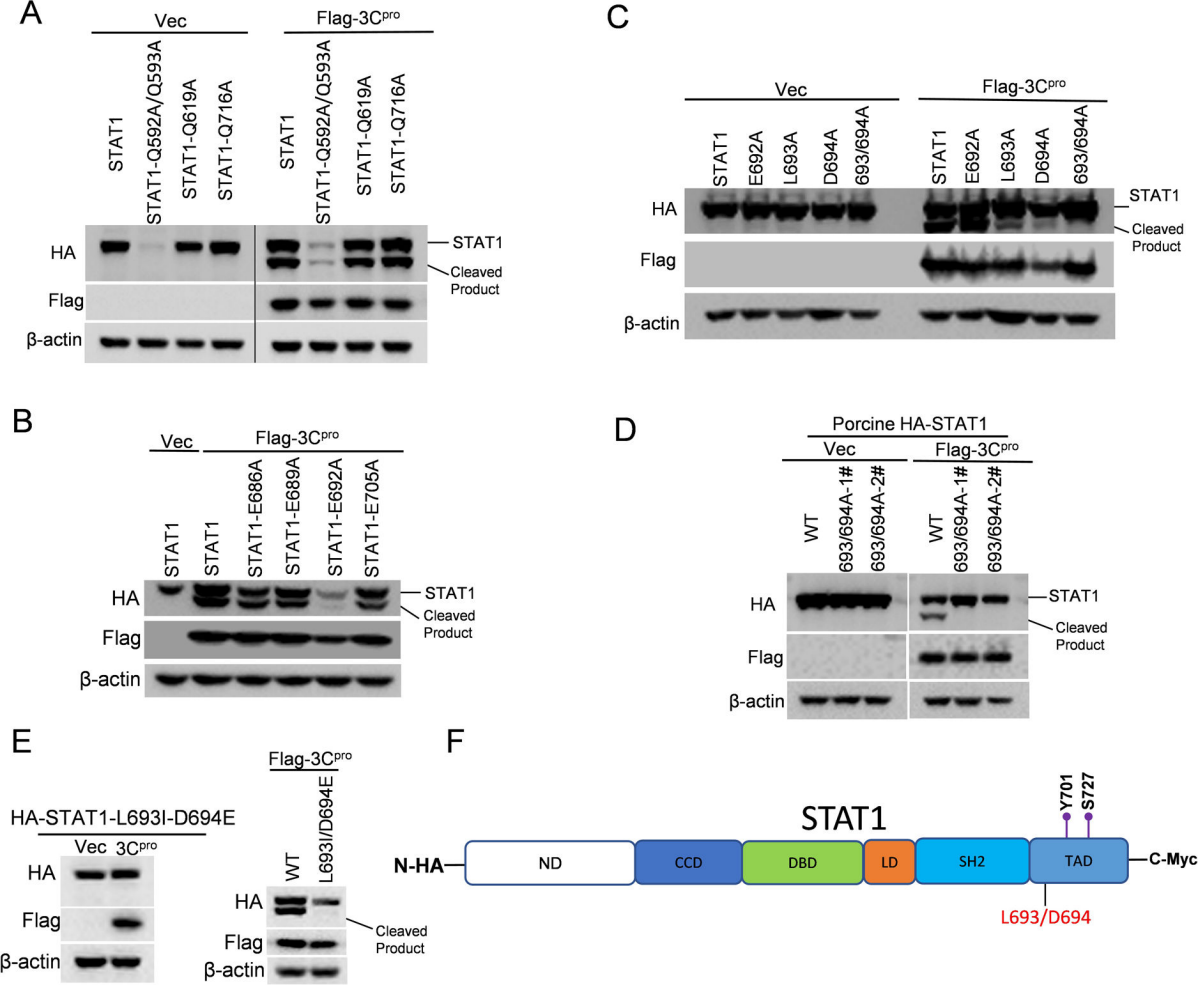
Identification of the SVA 3C^pro^ cleavage sites in human and porcine STAT1. (**A–C, E**) HEK-293T cells were transfected with human-STAT1 or the indicated STAT1 mutants expressing plasmids, along with empty vector or 3C^pro^ expressing plasmids for 24 h. The cleavage states of STAT1 and STAT1 mutants in the presence or absence of 3C^pro^ were analyzed by western blotting. (**D**) HEK-293T cells were transfected with plasmids encoding porcine-STAT1 or STAT1 mutant (1# and 2# represent two different clones of plasmid) and empty vector or SVA 3C^pro^ plasmid for 24 h. (**F**) Schematic diagram of STAT1. ND: N-terminal domain; CCD: coiled-coil domain; DBD: DNA-binding domain; LD: Linker domain; SH2: Src homology 2 domain; TAD: transactivation domain1. Y701/S727: Phosphorylation sites. L693/D694: SVA 3C^pro^ cleavage sites within human and porcine STAT1. All experiments were repeated three times.

We next sought to map the cleavage sites of STAT1 and STAT2 recognized by SVA 3C^pro^. Previous studies indicated that the preference substrate recognized by picornaviral 3C^pro^ was glutamine-glycine (Q-G) or glutamic acid-glutamine (E-Q) junctions (27). Given that the cleavage sites of 3C^pro^ within STAT1 and STAT2 were close to their C-termini, we initially analyzed the classical Q residues within the C-terminal STAT1 and STAT2. Because the cleaved-STAT1 fragment band was above 70kd, we individually substituted putative Q residues, specifically sites 592, 593, 619, or 716 sites at the C-terminus of STAT1 (human), with alanine (A) on the plasmids. The mutant was transfected with 3C^pro^ construct into HEK-293T cells for 24 h. Mutating these sites was not able to impact the cleavage of STAT1 by 3C^pro^ (Fig. 6A). We then chose E residue to analyze; putative E residues at the C-terminal of STAT1 (human) including the sites of 686, 689, 692, or 705 were individually mutated to Alanine (A) and then overexpressed with 3C^pro^. None of these sites were responsible for STAT1 cleavage (Fig. 6B). Previous studies showed that SVA 3C^pro^ exhibited low homology with other picornaviral 3C^pro^, which might result in a change in cleavage specificity (26). We reasoned that 3C^pro^ might recognize other residues on STAT1 that lead to cleavage. We then constructed a set of truncated STAT1 (human) by deleting the possible substrate recognition regions by 3C^pro^ (deleted region including amino acids 669–678, 679–688, 689–698, or 699–708). Notably, the deletion of amino acids 689–698 within STAT1 resulted in no cleavage products by 3C^pro^ (Fig. S3A). Based on this observation, we subsequently generated three truncated STAT1 (human) by deleting amino acid 689–691, 692–694, or 695–698 to deeply identify the substrate recognition region. No cleavage product was observed in the presence of truncated STAT1 with amino acid 692–694 deletion (Fig. S3B). We then focused on the amino acid 692–694 within STAT1 and constructed three single-site mutants (E692A, L693A, and D694A), and one double-site mutants (L693A/D694A). We eventually identified that L693-D694 motif serves as the cleavage site within STAT1 by 3C^pro^ (Fig. 6C). By comparing the amino acid sequence of human and porcine STAT1 (Fig. S3F), we identified that the corresponding residue as L693/D694 in porcine STAT1; mutating these two residues to A completely disrupted the cleavage of SVA 3C^pro^ on it (Fig. 6D). To eliminate the possibility that alanine substitutions might affect the local secondary structure or buries scissile bonds of STAT1, we constructed a new mutant in which Leucine 693 and Aspartic acid 694 were conservatively replaced by isoleucine and glutamic Acid, respectively, which have similar properties. The mutant (L693I-D694E) remained resistant to the SVA 3C^pro^ cleavage (Fig. 6E). We then predicted structures of WT STAT1, STAT1 (L693A-D694A) and STAT1 (L693I-D694E) using Alphafold on Colab (28) (Fig. S4A). It is notable that L693-D694 is located in a loop region. The loop region (Y681-706I) of WT STAT1 and mutated STAT1 were superimposed to calculate root mean square deviation (RMSD) with the values of 1.82  Å (WT versus L693A/D694A STAT1) and 1.23  Å (WT versus L693I-D694E STAT1); these values indicate amino acid substitution had a minor effect on the local secondary structure. Overall, the results reveal that L693 and D694 of STAT1 are critical for the SVA 3C^pro^-mediated cleavage.

**Fig 7:**
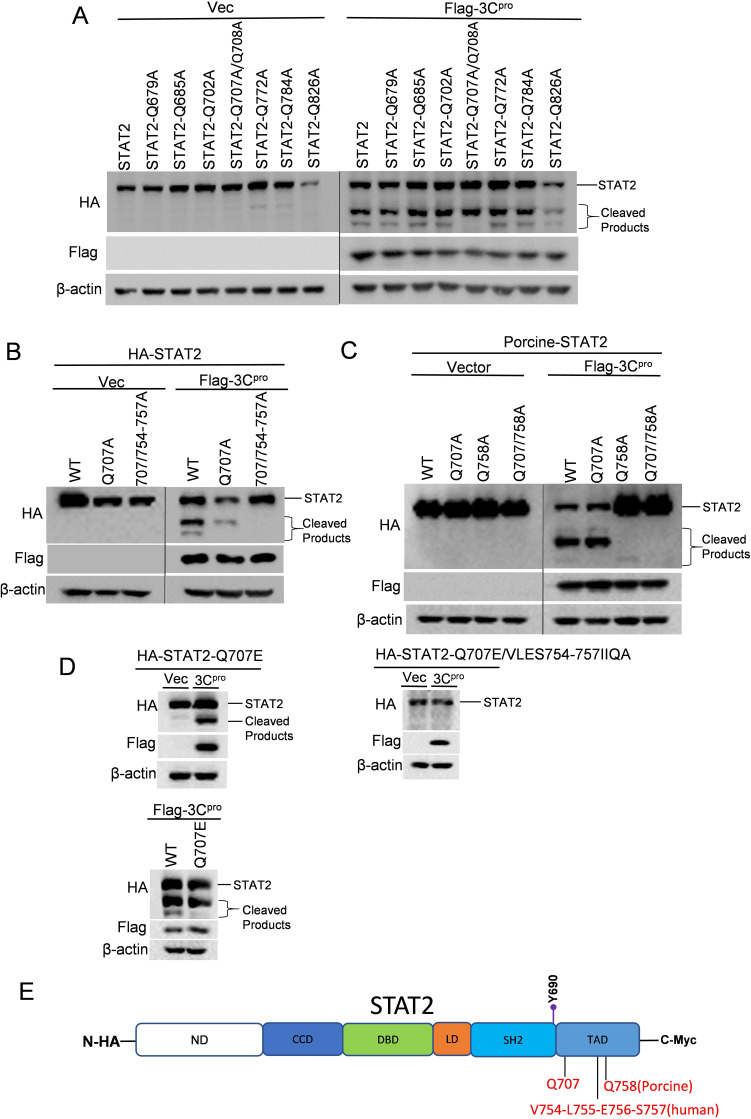
Identification of the cleavage sites in human and porcine STAT2 by SVA 3Cpro. (**A, B, D**) HEK-293T cells were transfected with human-STAT2 or indicated STAT2 mutants expressing plasmids, along with empty vector or 3C^pro^ expressing plasmids for 24 h. The cleavage states of STAT2 and STAT2 mutants in the presence or absence of 3C^pro^ were determined using western blotting. (**C**) HEK-293T cells were transfected with plasmids encoding porcine-STAT2 or indicated STAT2 mutant and vector or SVA 3C^pro^ plasmid for 24 h. (**E**) Schematic diagram of STAT2. ND: N-terminal domain; CCD: coiled-coil domain; DBD: DNA-binding domain; LD: Linker domain; SH2: Src homology 2 domain; TAD: transactivation domain1. Y690: Phosphorylation sites. Q707: SVA 3C^pro^ cleavage sites within human and porcine STAT1. Q758: Cleavage sites within porcine STAT2 by SVA 3C^pro^; V754-L755-E756-S757: Cleavage sites within human STAT2 by SVA 3C ^pro^. All experiments were repeated three times.

We used a similar approach to identify SVA 3C^pro^ substrate recognition sites in STAT2. In terms of the molecular weight of the two cleavage products, we initially selected eight Q residues within the region of amino acid 679–826 within STAT2 (human) to identify the cleavage site near the C-terminal. Seven single mutants (Q679A, Q685A, Q702A, Q772A, Q784, Q779A, and Q826A) and one double mutant (Q707A/Q708A) of STAT2 (human) were constructed and tested. We found that cleavage bands with lower molecular weight disappeared when co-expressed the double mutant (Q707A/Q708A) with SVA 3C^pro^ (Fig. 7A; Fig. S3C (the third lane for Q779A)). We then created two single mutants, with either Q707A or Q708A and determined that Q707 serves as the recognition site within STAT2 (human) for SVA 3C^pro^ cleavage (Fig. S3C). To identify the responsible residues for the cleavage product with higher molecular weight (cleavage site close to C-terminal), we generated a series of truncated STAT2 (human) by deleting ten residues each, including amino acids 721–730, 731–740, 741–750, and 751–760. The truncated STAT2 with deletion of amino acids 751–760 was resistant to cleavage, implying the second cleavage site is located within this region (Fig. S3D). Based on this region (amino acids 751–760), we created other three truncated STAT2, with mutants of amino acids 751–753, 754–756, and 757–760, and found residues with amino acids 754–757 serve as the second cleavage sites within STAT2 (human) (Fig. S3E). Remarkably, the amino acids within this region are Val-Leu-Glu-Ser motifs, which differ from the canonical substrate recognition patterns used by picornavirus 3C^pro^. We then mutated Q707 and 754-VLES-757 to A in the STAT2, and as expected, STAT2 with these mutations is completely resistant to SVA 3C^pro^ cleavage (Fig. 7B). By analyzing the amino acid sequence alignment between human and porcine STAT2 (Fig. S3G), we substituted the corresponding residue Q707 and Q758 in porcine STAT2 with A. The result showed that mutation in these two residues rendered porcine STAT2 resistant to SVA 3C^pro^ cleavage (Fig. 7C). To avoid the potential effects of alanine substitutions on the local secondary structure, similar to STAT1, we introduced conservative replacements to generate a human STAT2 mutant with Q707E and 754-VLES-757/754-IIQA-757 mutations instead of Q707A and 754-VLES-757/754-AAAA-757. These new mutations within STAT2 maintained its resistance to 3C^pro^ cleavage (Fig. 7D). We predicted the structures of WT-STAT2, Q707A/754-VLES-757/754-AAAA-757-STAT2 and Q707E/754-VLES-757/754-IIQA-757-STAT2 using Alphafold2 on Colab (28) (Fig. S4B) and observed that these cleavage sites, Q707 and 754-VLES-757, are also located on the loop. We then superimposed the residues (E705-P710, L751-E760) surrounding these two cleavage sites of WT-STAT2 and mutated STAT2, and the RMSD values (E705-P710: WT versus AAAA = 0.32 Å, WT versus IIQA = 0.72 Å; L751-E760: WT versus AAAA = 2.4 Å, WT versus IIQA = 2.5 Å) indicated alanine and conservative replacements do not significantly impact the local secondary structure.

Collectively, these data suggest that STAT2 contains two cleavage sites, with Q707 conserved in both porcine and human STAT2, while different cleavage patterns are observed at another site. In human STAT2, the V754-L755-E756-S757 motif is present, whereas porcine STAT2 contains Q758.

### Cleavage of STAT1/2 by SVA 3C^pro^ impairs the IFN signaling transduction

**Fig 8:**
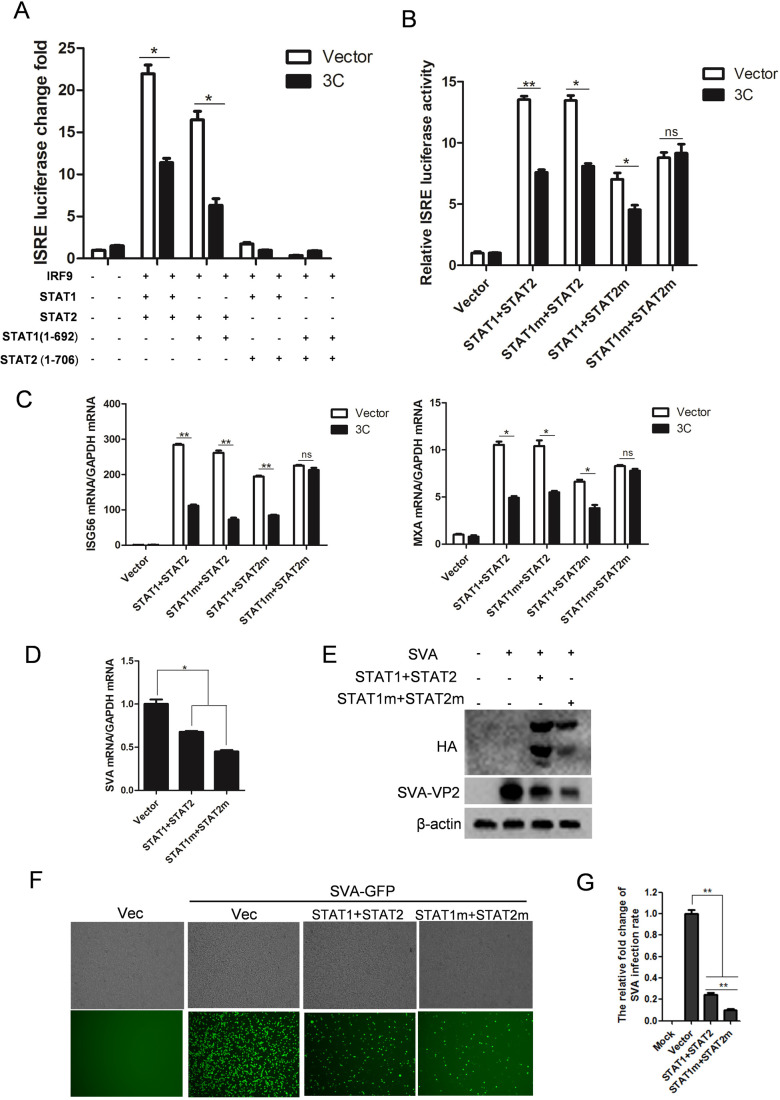
The impact of cleaved STAT1/2 and mutated STAT1/2 on the IFN signaling pathway. (**A**) HEK-293T cells were transfected with the indicated plasmids shown in (**A**) and ISRE reporter and PRL-TK plasmid. After 24 h, cell lysates were harvested to measure luciferase activity. White bar: empty vector transfection; Black bar: 3C^pro^ plasmid transfection. (**B**) HEK-293T cells were transfected with empty vector or 3C^pro^ expressing plasmids, and empty vector, STAT1 + STAT2, STAT1m + STAT2, STAT1 + STAT2 m, or STAT1m + STAT2 m expressing plasmids, together with ISRE reporter plasmids, IRF9 expressing plasmids and PRL-TK plasmids for 24 h, then the luciferase activity was measured by dual luciferase assay. (**C**) Transfections were performed similarly to those in (**B**). At 24 h post-infection, mRNA levels of *ISG56* and *MXA* were measured by qPCR. (**D and E**) HEK-293T cells were transfected with empty vector, STAT1 + STAT2, or STAT1m + STAT2 m expressing plasmids for 24 h, followed by SVA infection for 12 h. The levels of mRNA and protein of SVA were determined by qPCR (**D**) and Western blotting (**E**), respectively. (**F and G**) HEK-293T cells were transfected with empty vector, STAT1 + STAT2, or STAT1m + STAT2 m expressing plasmids for 24 h, followed by SVA-GFP infection for 12 h. The fluorescent intensity of eGFP was visualized and quantified visually. All experiments were repeated three times.

To investigate the effect of STAT1 and STAT2 cleavage by SVA 3C^pro^ in the JAK-STAT signaling pathway, we constructed two truncated constructs, STAT1 (1-692) and STAT2 (1-706), with lengths equivalent to the cleaved products. We then examined ISRE promoter activity induced by ISGF3 when overexpressed either of them with WT-STAT1 or WT-STAT2, as well as each other, in the presence or absence of 3C^pro^. Co-expressing STAT2 (1-706) with either STAT1 (1-692) or WT-STAT1 failed to activate ISRE promoter, regardless of the presence or absence of 3C^pro^ (Fig. 8A; Fig. S4C). The co-expression of STAT1 (1-692) and WT-STAT2 still could induce ISRE promoter, albeit at reduced levels compared to co-expression of WT STAT1 and STAT2 (Fig. 8A). However, the ISRE promoter activities activated by them were decreased when 3C^pro^ was expressed (Fig. 8A). These data suggest that the cleavage of STAT1 and STAT2 by 3C^pro^ impairs the activation of IFN signaling pathway.

We then tested whether STAT1 and STAT2, lacking SVA 3C^pro^ cleavage sites, are still capable of activating JAK-STAT pathway transduction. To do this, ISRE-luciferase and pRL-TK reporter plasmid were co-transfected with plasmid encoding wide-type STAT1 or STAT2 and their respective mutated forms, mSTAT1 or mSTAT2, along with SVA 3C^pro^ construct or vector, as well as IRF9 plasmid, into HEK-293T cells. Co-expression of these two STAT mutants with their corresponding WT-STAT could induce the ISRE-promoter activity, although this induction was inhibited in the presence of 3C^pro^ (Fig. 8B; Fig. S4D). Notably, the ISRE-promoter activity unregulated by mSTAT1 and mSTAT2 was resistant to the antagonistic effect of 3C^pro^ (Fig. 8B). Similar results were observed when assessing the expression of ISGs, ISG56, and MxA (Fig. 8C). To assess the effect of mSTAT in the viral infection context, we transfected HEK-293T cells with WT STAT1/STAT2 or its mutants for 24 h, followed by SVA infection for 12 h. As expected, overexpression of STAT1 and STAT2 suppressed SVA infection (Fig. 8D and E); the levels of viral mRNA and protein were significantly decreased in cells expressing WT STAT1/STAT2 or mutated STAT1/STAT2. Particularly, STAT1/STAT2 mutants showed a stronger antiviral activity than WT STAT1/STAT2. We obtained a similar result when measuring the replication of SVA-GFP virus in the context of overexpressing these STAT mutants (Fig. 8F and G). Taken together, these data reveal that STAT1/2 with disrupted 3C^pro^ cleavage sites are resistant to the antagonistic effect of 3C^pro^, thereby inducing IFN signaling pathway for inhibiting SVA infection.

## DISCUSSION

In this study, we reveal that SVA 3C^pro^ suppresses the IFN-induced response by cleaving and degrading STAT1 and STAT2; this observation was confirmed in the context of virus infection. We also confirmed that among all the interferon response antagonistic viral proteins of SVA (VP2, 2AB, 2B, 3C^pro^, and 3D^pol^ were identified by luciferase assay in Fig. 2A), only 3C^pro^ specifically impacts STAT1 and STAT2, while the others cannot. This indicates that the observed phenotype of STAT1 and STAT2 cleavage and degradation in SVA infection is caused by 3C^pro^. The cleavage required the protease activity of 3C^pro^, as well as the direct interactions between 3C^pro^ and STAT1 or STAT2. Meanwhile, we noted that Z-VAD-FMK, a pan-caspase inhibitor, partially reverses the degradation of STAT1 and STAT2 caused by 3C^pro^, which suggests 3C^pro^ activates apoptotic pathway to intensify their degradation, supporting previous reports that indicated the crucial role of 3C^pro^ in triggering cellular apoptosis during SVA infection (7, 8). The cleavage sites of human and porcine STAT1 by SVA 3C^pro^ were mapped to residues L693/D694. In both human and porcine STAT2, Q707 served as a cleavage site, resulting in a shorter cleaved product. However, distinct cleavage sites, V754-L755-E756-S757 motif in humans and Q758 in porcine STAT2, were responsible for the longer cleaved product. These cleavage patterns partially differ from the preference cleavage sites by picornaviral 3C^pro^, typically Q-G or E-Q junctions, suggesting the broader substrate recognition capability of SVA 3C^pro^. Previous studies have shown that low sequence identity and structural difference were found between SVA 3C^pro^ and other picornaviral 3C^pro^ (26), implying that SVA 3C^pro^ might possess substrate recognition abilities distinct from those of its counterparts. Our finding identifies novel cleavage sites on STAT1 and STAT2 by SVA 3C^pro^ and provides evidence that diverse recognition patterns exist in picornaviral 3C^pro^.

Picornavirus utilizes various strategies to dysregulate the IFN-mediated transduction of the JAK-STAT pathway to promote viral infection and transmission (16). Among these strategies, 3C^pro^ of FMDV was found to induce the degradation of karyopherin α1, which is critical for STAT1 and STAT2 nuclear translocation (17). In addition, EV71 3C^pro^ has been shown to mediate the cleavage of IRF9, thereby inhibiting the ISGF3 nuclear translocation (18). Here, we found that unlike FMDV and EV71, SVA 3C^pro^ can target two distinct molecules, STAT1 and STAT2, resulting in their cleavage and degradation. In the presence of Z-VAD-FMK, the degradation of STAT1 and STAT2 caused by 3C^pro^ was partially inhibited, while cleavage remained unaffected, indicating 3C^pro^ mediated the degradation of STAT1 and STAT2 also occurs in a caspase-dependent manner. However, inactivating the catalytic sites of SVA 3C^pro^ simultaneously inhibited its ability to cleave and degrade STAT1 and STAT2. These observations suggest that the cleavage of STAT1 and STAT2 is completely dependent on the protease activity of 3C^pro^, rather than caused by any caspase enzymes. Furthermore, the protease activity of 3C^pro^ is also crucial for the caspase-pathway-mediated STAT1 and STAT2 degradation. Prior studies have shown that SVA 3C^pro^ has the ability to induce apoptosis, with its protease activity being essential for this process (8), which supports current findings regarding the critical role of 3C^pro^’s protease activity in mediating the caspase-induced degradation of STAT1 and STAT2. Given the pivotal roles of STAT1 and STAT2 in the IFN response signaling pathway, viruses have evolved various means to subvert their functions for establishing infection. The cleavage or degradation of STATs also has been reported in other viruses. For instance, two flaviviruses, ZIKA and DENV, can employ their NS5 proteins to bind with STAT2 and promote its degradation *via* the proteasome pathway (29, 30). V protein of Simian virus 5 degrades STAT1 in a proteasome-dependent manner (31). Porcine deltacoronavirus utilizes its 3C-like protease, nsp5, to target STAT2 for protease activity-mediated cleavage (32). Strikingly, our observations reveal that SVA 3C^pro^ not only cleaves both STAT1 and STAT2 but intensified their degradation *via* the caspase-mediated pathway. However, the precise mechanism through which 3C^pro^-induced caspase-pathway activation contributes to the degradation of STAT1 and STA2 remains unclear. STAT1 is cleaved by activated caspase-3 during apoptosis (33, 34). Previous study showed that African swine fever virus infection could induced apoptosis that triggered the activation of caspase-3, leading to STAT1 cleavage (35). We found that the SVA 3C^pro^-induced apoptosis is not responsible for STAT1 cleavage but does cause the degradation of STAT1, thus suggesting an alternative mechanism was used by 3C^pro^. One plausible mechanism by which 3C^pro^ degrades STAT1 and STAT2 might involve the downregulation of their protein synthesis, which is usually accompanied by apoptosis (36). In some cases, eukaryotic initiation factors have been reported to be modified and lose their function during apoptosis, for example, eIF4GI and eIF4GII are known to be cleaved by caspase-3, and eIF4E is de-phosphorylated, all of which can inhibit protein translation (37–41). Our observations indicated that the expression of both STAT1 and STAT2 are decreased in the presence of 3C^pro^, not only STAT1. This implies that their degradation might be involved in the inhibition of protein synthesis.

Classically, cleavage sites by picornaviral 3C^pro^ on the viral polyprotein and host cellular proteins are typically Q-G or E-Q junctions (27). However, intriguingly, different from other picornaviruses, our investigation revealed that, besides residue Q, SVA 3C^pro^ could recognize non-canonical substrates on STAT1 and STAT2 for cleavage. The cleavage patterns in human and porcine STAT1 were identical, with residues L693/D694 identified in them. Meanwhile, we confirmed two cleaved positions in STAT2; Q707 was identical in both human and porcine STAT2, while the second site exhibited distinct cleavage patterns. In human STAT2, a four amino acids sequence (754-VLES-757) serves as the second region critical for SVA 3C^pro^ cleavage, with any mutations within this sequence abolishing the cleavage. However, a single residue, Q758 within porcine STAT2, was recognized by SVA 3C^pro^ as a second site. These findings provide evidence to underpin the prior notion that SVA 3C^pro^ might recognize a wider range of substrates compared to other picornaviral 3C^pro^ (26). Due to the low sequence similarity of SVA 3C^pro^ with counterparts in other picornaviruses, the structural comparison revealed that slight differences in substrate subsites within SVA 3C^pro^ compared to others (26). Specifically, SVA 3C^pro^ contains wider S1′ and S4 pockets in comparison to other 3C^pro^, which might result in various substrate recognition patterns (26). Prior studies have shown that SVA 3C^pro^ utilizes its protease ability to cleave host factors, and it is noteworthy that all the identified cleavage sites are Q or E residues (5, 6); our results, which identify non-canonical residues recognized by SVA 3C^pro^ for cleavage, have expanded our understanding of cleavage pattern mediated by picornaviral 3C^pro^.

It is widely known that STAT1 and STAT2 possess multiple functional conserved domains, as depicted in Fig. 6F and 7E. A completed C-terminal transactivation domain (TAD) within STAT1 is critical for inducing the expression of antiviral genes (11, 42, 43). Our results reveal that SVA 3C^pro^ cleaves STAT1 at L693/D694, resulting in a truncated STAT1 (1-692) lacking a significant portion of the TAD, which lost all the activity when overexpressed with truncated STAT2 (1-706), another cleaved product by 3C^pro^. Notably, the truncated STAT1 (1-692) lacks two critical phosphorylated sites, tyrosine 701 and serine 727, which are essential for the activation of STAT1 upon interferon stimulation (44). The cleavage occurred in STAT2 produced two truncated forms: one in human or porcine STAT2 (1-707) and another in human STAT2 (1-754) or porcine STAT2 (1-758). We provided evidence that truncated STAT2 (1-706) was not able to activate ISRE promoter activity when co-transfected with STAT1 (1-692). Another study reported that Q758 is also targeted by Porcine deltacoronavirus nsp5 for mediating the cleavage of porcine STAT2 (32). They demonstrated that truncated STAT2 (1-758) significantly impaired JAK-STAT signaling pathway induction, implying that truncated STAT2 (1-754) and (1-758) produced by SVA 3C^pro^ also lose the ability to activate JAK-STAT pathway during the SVA infection.

**Fig 9:**
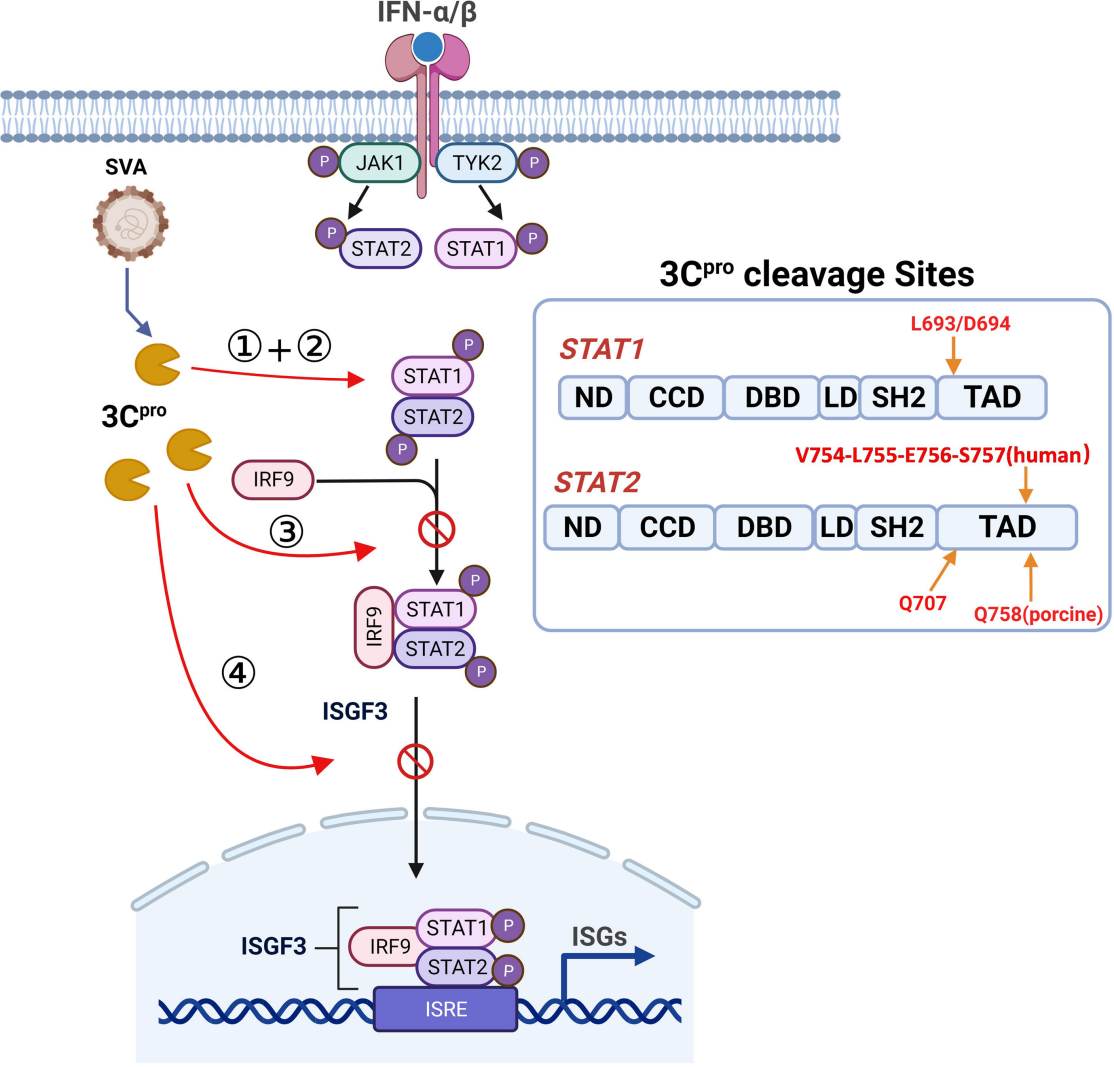
Putative model of how SVA 3C^pro^ negatively mediates type I IFN response. When SVA infected host cells, 3C^pro^ interacts with STAT1 and STAT2 and utilizes its protease activity to cleave STAT1 and STAT2 (①), resulting in reduced phosphorylation of STAT1 and STAT2 (②), which further disrupting the formation (③) and nuclear translocation (④) of ISGF3 complex, consequently diminishing IFN-mediated antiviral response, ultimately benefiting SVA infection. The cleavage sites in STAT1 and STAT2 by SVA 3C^pro^ are shown at right inset.

Collectively, our results unveil another function of SVA 3C^pro^ as an IFN antagonist, specifically its role in inhibiting JAK-STAT pathway transduction (as shown in Fig. 9). SVA 3C^pro^ utilizes its protease activity to cleave STAT1 and STAT2, resulting in reduced phosphorylation of STAT1 and STAT2, which further disrupting the formation and nuclear translocation of ISGF3 complex, consequently diminishing IFN mediated antiviral response, ultimately benefiting SVA infection.

## MATERIALS AND METHODS

### Cells and viruses

Human embryonic kidney 293T (HEK-293T) cells, porcine kidney PK-15, U3A cells (STAT1-null), and U6A cells (STAT2-null) were maintained in Dulbecco’s Modified Eagle Media (DMEM, VivaCell) containing 10% fetal bovine serum (FBS) and cultured at 37°C in a humidified incubator with 5% CO_2_. SVA strain CH-FJ-2017 (GenBank accession number KY747510) was used in the present study (45). U3A and U6A cells were gifts from Dr. Jinbo Yang (Ocean University of China, Qingdao, China).

### Plasmids, antibodies, and reagents

The mammalian expression constructs encoding human and porcine STAT1 or STAT2 were generated by PCR amplification of the corresponding CDS of STAT1 and STAT2 from HEK-293T and PK-15 cells, respectively. The amplified fragments were inserted into a pcDNA3.1 myc-His A vector (Invitrogen) or a modified pcDNA3.1 myc-His A vector containing an N-terminal HA tag. The plasmids of SVA 3C^pro^ or the 3C^pro^ mutants (H48A, C160A, and H48A/C160A) were constructed as previously described (46). The luciferase reporter plasmid harboring the IFN-stimulated response element (ISRE) (pISRE-Luc) and the reference plasmid pRL-TK were kindly provided by Dr. Hongbing Shu (Wuhan University, China) (47, 48). All the constructs expressing mutants of STAT1 and STAT2 in this study were made using site-directed mutagenesis PCR. Sequences of all constructs were verified prior to use. Primary antibodies used in this study included: anti-Myc, anti-HA, anti-Flag, and anti-β-actin mouse mAb (Sigma-Aldrich); anti-STAT1, anti-STAT2, anti-pSTAT1, and anti-pSTAT2 rabbit mAb (Cell signaling technology). Goat anti-mouse or rabbit IgG (H + L) secondary antibodies were purchased from Biodragon company. Anti-SVA 3C^pro^ rabbit pAb was made in-house. Recombinant human alpha-interferon (IFN-α) and beta-interferon (IFN-β) were purchased from PBL assay Science. Recombinant porcine alpha-interferon (IFN-α) and beta-interferon (IFN-β) were made in-house.

### Dual-luciferase reporter assay

HEK-293T cells (5 × 10^4^/well) were seeded onto 24-well plates, and cells were transfected with pISRE-Luc plasmid (50 ng), pRL-TK plasmid (5 ng), and the indicated plasmids using jetPRIME DNA transfection reagent for the specified duration. IFN-α (1,000 U/mL) and IFN-β (1,000 U/mL) were used in the indicated experiments. Cell lysates in 1× passive lysis buffer were utilized to measure luciferase activity using a Dual-Luciferase Reporter assay system, following the manufacturer’s protocol (Promega). Data represent the relative firefly luciferase activity normalized to the Renilla luciferase activity.

### Co-immunoprecipitation and western blotting analysis

HEK-293T cells grown in 10 cm dishes were transfected with the indicated plasmids for 24 h or infected with SVA for 12 h, and the cells were then lysed in the ice-cold NP-40 lysis buffer for immunoprecipitation described previously (49, 50). Briefly, the cell lysates were immunoprecipitated with 50% (vol/vol) slurry of GammaBind G Plus-Sepharose (GE Health Care Life Sciences) binding with indicated primary antibodies at 4°C. IP and input samples were then subjected to western blotting analysis. Prepared samples were mixed with the SDS loading buffer and denatured at 95°C for 5 min. The boiled samples were subsequently centrifugated at 12,000 rpm for 15 min to remove cell debris. The supernatants were electrophoresed in 8% or 10% polyacrylamide gels and transferred onto BioTrace NT nitrocellulose membrane, followed by immunoblotting with the indicated antibodies as described previously (51).

### RNA extraction and real-time qPCR

Trizol reagent (moibio) was used to extract total RNAs of cells. The reverse transcribed complementary DNA (cDNA) was synthesized using HiScript II Q RT SuperMix for qPCR (Vazyme) following the manufacturer’s protocol. The cDNAs were used for analyzing indicated target genes by using ChamQ Universal SYBR qPCR Master Mix (Vazyme) and gene-specific primers. The sequences of qPCR primers used in this study are listed in Table 1. The relative transcript levels of the indicated genes were calculated using the 2^ΔΔCT^ method with the glyceraldehyde-3-phosphate dehydrogenase (GAPDH) as a housekeeping gene.

**TABLE 1:** Primers of target genes for qPCR used in this study

Target genes	Primers	Sequences (5′−3′)
SVA	Forward	AGAATTTGGAAGCCATGCTCT
	Reverse	GAGCCAACATAGAAACAGATTGC
h-ISG15	Forward	TGGACAAATGCGACGAACC
	Reverse	CCCGCTCACTTGCTGCTT
h-GBP1	Forward	CGAGGGTCTGGGAGATGTAG
	Reverse	TAGCCTGCTGGTTGATGGTT
h-ISG54	Forward	ACGGTATGCTTGGAACGATTG
	Reverse	AACCCAGAGTGTGGCTGATG
h-ISG56	Forward	TAGCCAACATGTCCTCACAGAC
	Reverse	TCTTCTACCACTGGTTTCATGC
h-MxA	Forward	TCTTCATGCTCCAGACGTAC
	Reverse	CCAGCTGTAGGTGTCCTTG
h-GAPDH	Forward	CGGGAAGCTTGTGATCAATGG
	Reverse	GGCAGTGATGGCATGGACTG
p-IFI44L	Forward	TAGGATAGCAGGAGCCACA
	Reverse	TACGGATTTCTGAAACCAAGT
p-MXA	Forward	GGCGTGGGAATCAGTCATG
	Reverse	AGGAAGGTCTATGAGGGTCAGA
p-GAPDH	Forward	ACATGGCCTCCAAGGAGTAAGA
	Reverse	GATCGAGTTGGGGCTGTGACT

### SVA-eGFP infection

SVA-eGFP constructed by our laboratory (52) was used to determine the effects of STAT1 and STAT2 mutants on SVA. In brief, HEK-293T cells were transfected with STAT1/STAT2 or its mutants for 24 h, and the cells were inoculated with SVA-eGFP at an MOI of 0.1 for 12 h. Fluorescent intensity of eGFP was measured and visualized by inverted fluorescence microscopy (10× magnification using EVOS5000). The cells exhibiting fluorescence (indicating SVA infection) were visually quantified through manual counting to determine the infection state.

### Nuclear/cytoplasmic separation

The nuclear and cytoplasmic extracts were lysed and separated from the cells with a Nuclear/Cytosol Fractionation Kit (BioVision) according to the manufacturer’s protocols. Anti-β-actin and anti-histone Abs were used to assess the purity of the nuclear and cytoplasmic extracts, respectively. The prepared extracts were analyzed by Western blotting with the appropriate Abs.

### Statistical analysis

All data are represented as mean ± s.e.m from three independent experiments. Statistical analysis was performed with GraphPad Prism software (version 9) using an unpaired *t*-test with two-tailed *P*-values. A *P*-value less than 0.05 was considered to be statistically significant (*); a *P*-value less than 0.01 was deemed to be statistically highly significant (**).

## SUPPLEMENTAL MATERIAL

Supplemental material jvi.00585-24-s0001.pdf Figures S1 to S4.

ASM does not own the copyrights to Supplemental Material that may be linked to, or accessed through, an article. The authors have granted ASM a non-exclusive, world-wide license to publish the Supplemental Material files. Please contact the corresponding author directly for reuse.
