# Supplementary material for: Impaired interferon response in senecavirus A infection and identification of 3Cpro as an antagonist
Source: J Virol. 2024 Jun 13;98(7):e00585-24. doi: 10.1128/jvi.00585-24 (PMC11265225; doi:10.1128/jvi.00585-24)
Supplement: Supplemental material — Figures S1 to S4. [file jvi.00585-24-s0001.pdf]

Supplementary Materials

Impaired Interferon Response in Senecavirus A Infection and Identification of $3C^{pro}$ as an Antagonist

Xiangle Zhang[1,#], Pengfei Li[1,#,*,a], Wenzhe Chen[1], Shilei Zhang[1], Kangli Li[1], Yi Ru[1], Zhenxiang Zhao[1], Weijun Cao[1], Fan Yang[1], Hong Tian[1], Jianhong Guo[1], Jijun He[1], Zixiang Zhu[1,*], Haixue Zheng[1,*]

1. State Key Laboratory of Veterinary Etiological Biology, College of Veterinary Medicine, Lanzhou University, Lanzhou Veterinary Research Institute, Chinese Academy of Agricultural Sciences, Lanzhou 730000, China

Corresponding author:

* Address Correspondence to Haixue Zheng, zhenghaixue@caas.cn; Pengfei Li, lipengfei231x@foxmail.com; Zixiang Zhu, zhuzixiang@caas.cn.

[#] Xiangle Zhang and Pengfei Li contributed equally to this article.

[a] Present address: Pengfei Li, Department of Microbiology and Immunology, University of Iowa, Iowa City, IA 52242, USA

Keywords: Senecavirus A; 3C protease; Interferon response.

This PDF files includes:

Supplementary figures legends

Supplementary figures S1-S4

Figure.S1. The expression of SVA viral proteins in luciferase assay and qPCR of Fig. 2B. (A) The expression of individual SVA viral protein after transfection in Fig. 2A. (B-F) The expression of SVA $3C^{pro}$ in Fig.2B (B), Fig. 2C (C), Fig. 2D (D), Fig. 2E (E) and Fig.2F (F). (G) HEK-293T cells were infected with 0.05 or 0.1 MOI of SVA or without infection for 12 h. After 6h IFN-α treatment, mRNA levels of ISG54, ISG56 and MxA were measured by qPCR. All experiments were repeated at least three times. $^{*}p < 0.05$, $^{**} p < 0.01$.

Figure. S2. The expression of STAT1 and STAT2 in presence of individual SVA viral protein. (A-B) HEK-293T cells were transfected with empty vector or $3C^{pro}$ expressing plasmids, along with human-STAT1 (upper) or porcine-STAT1 (lower) expressing plasmids. The cells were harvested at 0h, 8h, 16h, 24h post transfection for subsequent analysis by Western blotting. (B) A similar process to that in (A) but using human-STAT2 plasmid (upper) or porcine-STAT2 plasmid (lower) instead. (C-D) HEK-293T cells were transfected with human-STAT1 (C) or human-STAT2 (D) expressing plasmids, along with empty vector or SVA viral protein expressing plasmids for 24h. The status of STAT1 and STAT2 under the expression of SVA VP2, 2AB, 2B, $3D^{pol}$, VP3 and VP1 were tested by Western blotting. (E) HEK-293T cells were inoculated with SVA at 1MOI, and viruses from supernatant were harvested at indicated time points. $TCID_{50}$ assays were performed to titrate the progeny viruses.

Figure.S3. The expression of STAT1 and STAT2 and their mutants in presence of SVA $3C^{pro}$. (A-C) HEK-293T cells were transfected with human-STAT1 or the indicated STAT1 mutants expressing plasmids, along with empty vector or $3C^{pro}$ expressing plasmids for 24h. (D-E) HEK-293T cells were transfected with plasmids encoding human-STAT2 or STAT2 mutants and empty vector or SVA $3C^{pro}$ plasmid for 24h. (1# and 2# represent two different clones of plasmids) (F-G) Sequence alignment of human and porcine STAT1 as well as human and porcine STAT2. Human-STAT1 (GU211347); Human-STAT2 (NM_005419); Porcine-STAT1 (NM_213769); Porcine-STAT2 (NM_213889);

Figure.S4. Structural comparison of predicted WT and mutated STATs. (A) The top-ranked structures of WT-human STAT1, STAT1 (L693A-D694A) and STAT1 (L693I-D694E) were displayed after prediction using Alphafold2 on Colab. Structures were modified using PyMoL, with Loop regions (Y681-706I) colored in orange. Resides at positions 693 and 694 are shown using spheres model. (B) Similar to (A), the structures of WT-human STAT2 and mutants with Q707E--754-VLES-757/754-IIQA-757 and Q707A--754-VLES-757/754-AAAA-757 were predicted by Alphafold2 on colab, and the top-ranked ones were displayed. Indicated residues on the loop (R701-P792, colored by yellow) are depicted using spheres model. (C-D). The expression level of individual plasmids in Fig.8A (C) and Fig.8B (D) was measured by western blotting.

Fig.S1

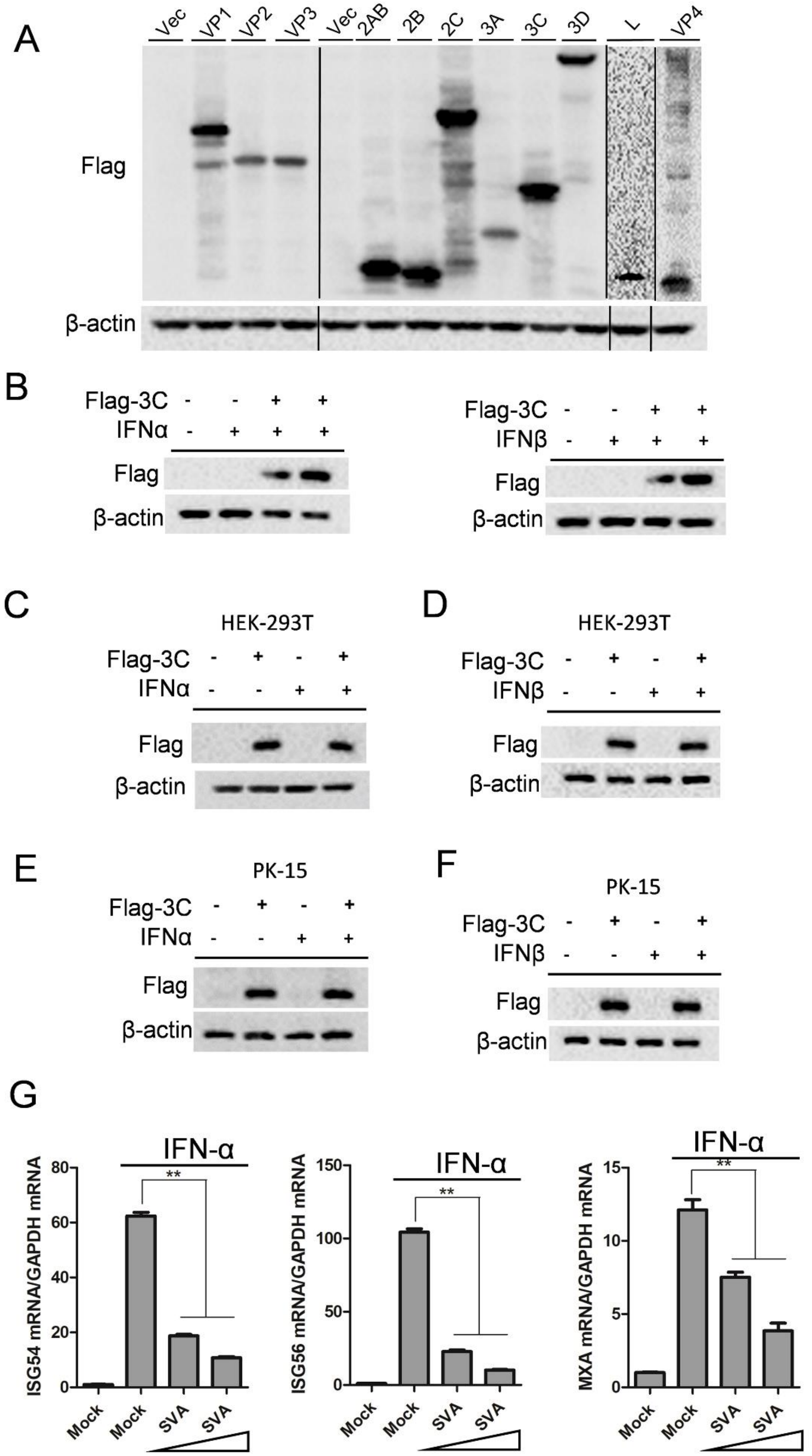
A
Vec
VP1
VP2
VP3
Vec
2AB
2B
2C
3A
3C
3D
L
VP4
Flag
β-actin
B
Flag-3C
IFNα
Flag
β-actin
Flag-3C
IFNβ
Flag
β-actin
C
HEK-293T
Flag-3C
IFNα
Flag
β-actin
D
HEK-293T
Flag-3C
IFNβ
Flag
β-actin
E
PK-15
Flag-3C
IFNα
Flag
β-actin
F
PK-15
Flag-3C
IFNβ
Flag
β-actin
G
IFN-α
ISG54 mRNA/GAPDH mRNA
ISG56 mRNA/GAPDH mRNA
MXA mRNA/GAPDH mRNA
Mock
Mock
SVA
SVA

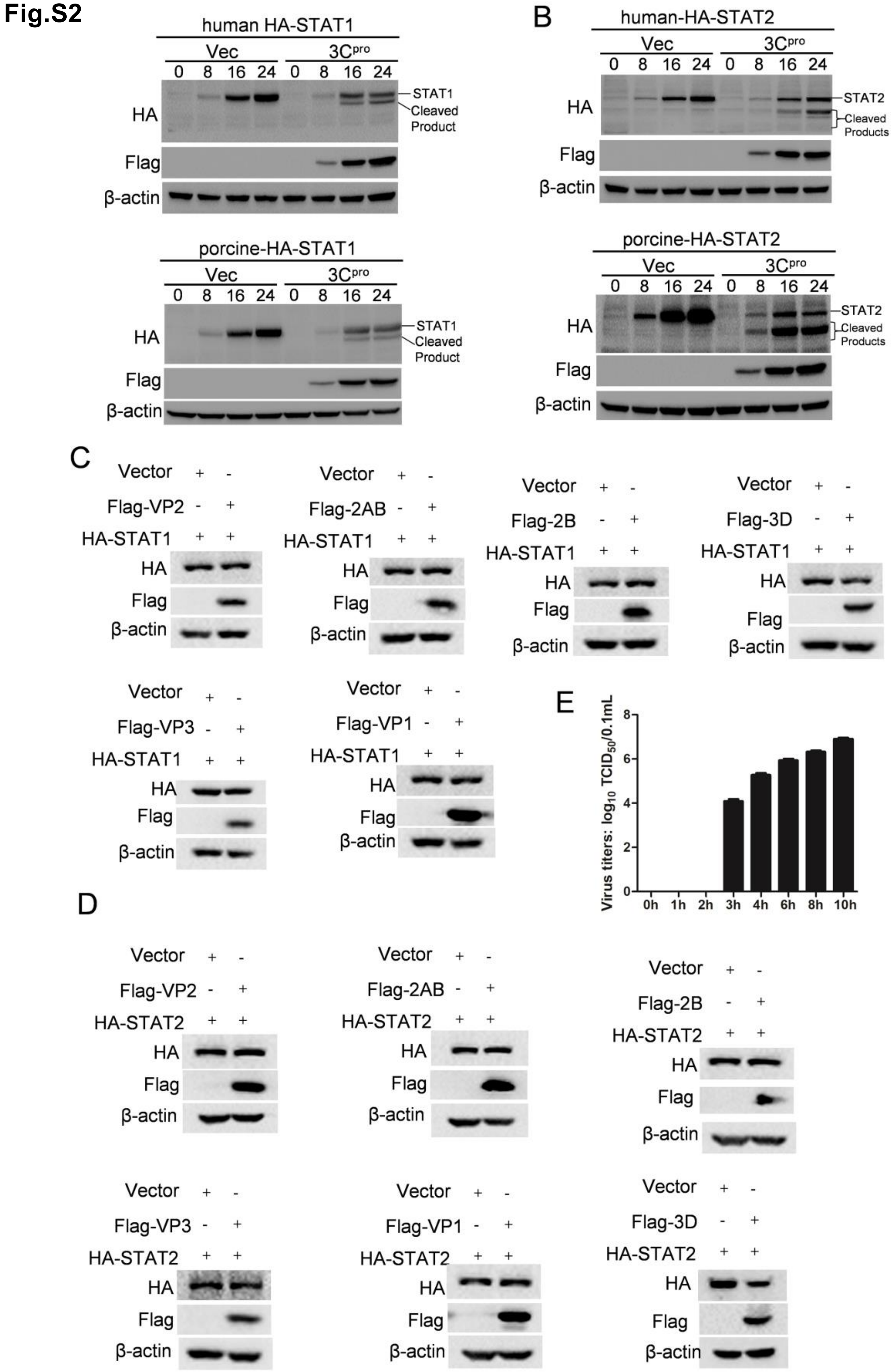
Fig.S2
human HA-STAT1
Vec
3Cpro
0 8 16 24 0 8 16 24
HA
Flag
β-actin
STAT1
Cleaved Product
porcine-HA-STAT1
B
human-HA-STAT2
STAT2
Cleaved Products
porcine-HA-STAT2
C
Vector
Flag-VP2
Flag-2AB
Flag-2B
Flag-3D
Flag-VP3
Flag-VP1
HA-STAT1
E
Virus titers: log10 TCID50/0.1mL
0h 1h 2h 3h 4h 6h 8h 10h
D
HA-STAT2

Fig.S3

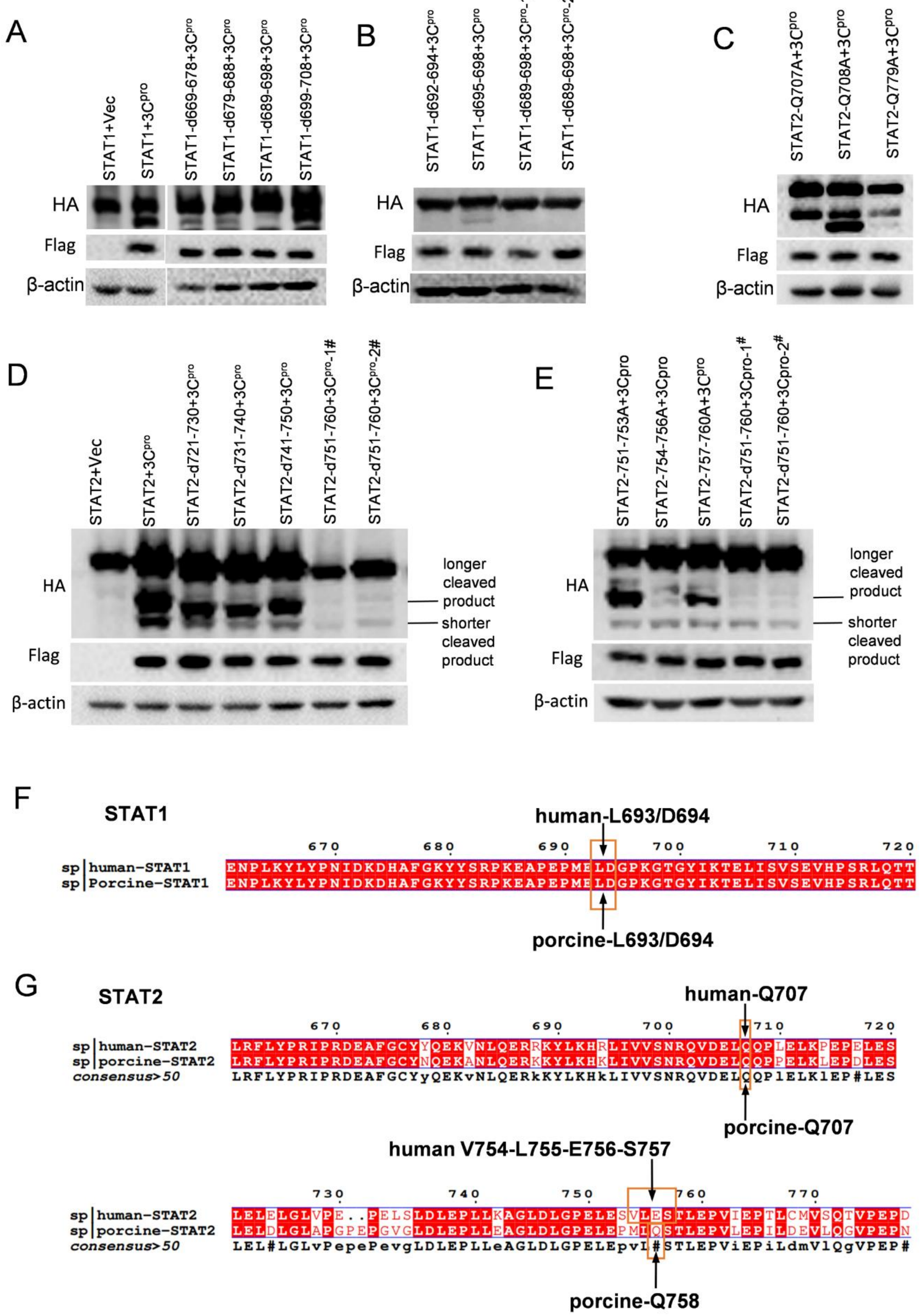
A
STAT1+Vec
STAT1+3Cpro
STAT1-d669-678+3Cpro
STAT1-d679-688+3Cpro
STAT1-d689-698+3Cpro
STAT1-d699-708+3Cpro
HA
Flag
β-actin
B
STAT1-d692-694+3Cpro
STAT1-d695-698+3Cpro
STAT1-d689-698+3Cpro-1#
STAT1-d689-698+3Cpro-2#
HA
Flag
β-actin
C
STAT2-Q707A+3Cpro
STAT2-Q708A+3Cpro
STAT2-Q779A+3Cpro
HA
Flag
β-actin
D
STAT2+Vec
STAT2+3Cpro
STAT2-d721-730+3Cpro
STAT2-d731-740+3Cpro
STAT2-d741-750+3Cpro
STAT2-d751-760+3Cpro-1#
STAT2-d751-760+3Cpro-2#
HA
Flag
β-actin
longer cleaved product
shorter cleaved product
E
STAT2-751-753A+3Cpro
STAT2-754-756A+3Cpro
STAT2-757-760A+3Cpro
STAT2-d751-760+3Cpro-1#
STAT2-d751-760+3Cpro-2#
HA
Flag
β-actin
longer cleaved product
shorter cleaved product
F
STAT1
human-L693/D694
670 680 690 700 710 720
sp|human-STAT1 ENPLKYLYPNIDKDHAFGKYYSRPKEAPEPMELDGPKGTGYIKTELISVSEVHPSRLQTT
sp|Porcine-STAT1 ENPLKYLYPNIDKDHAFGKYYSRPKEAPEPMELDGPKGTGYIKTELISVSEVHPSRLQTT
porcine-L693/D694
G
STAT2
human-Q707
670 680 690 700 710 720
sp|human-STAT2 LRFLYPRIPRDEAFGCYYQEKVNLQERRKYLKHRLIVVSNRQVDELQQPLELKPEPELES
sp|porcine-STAT2 LRFLYPRIPRDEAFGCYNQEKANLQERKKYLKHKLIVVSNRQVDELQQPPELKLEPDLES
consensus>50 LRFLYPRIPRDEAFGCYyQEKvNLQERkKYLKHkLIVVSNRQVDELQQPlELKlEP#LES
porcine-Q707
human V754-L755-E756-S757
730 740 750 760 770
sp|human-STAT2 LELELGLVPE..PELSLDLEPLLKAGLDLGPELESVLESTLEPVIEPTLCMVSQTVPEPD
sp|porcine-STAT2 LELDLGLAPGPEPGVGLDLEPLLEAGLDLGPELEPMIQSTLEPVLEPILDEVLQGVPEPN
consensus>50 LEL#LGLvPepePevgLDLEPLLeAGLDLGPELEpvI#STLEPViEPiLdmVlQgVPEP#
porcine-Q758

Fig.S4

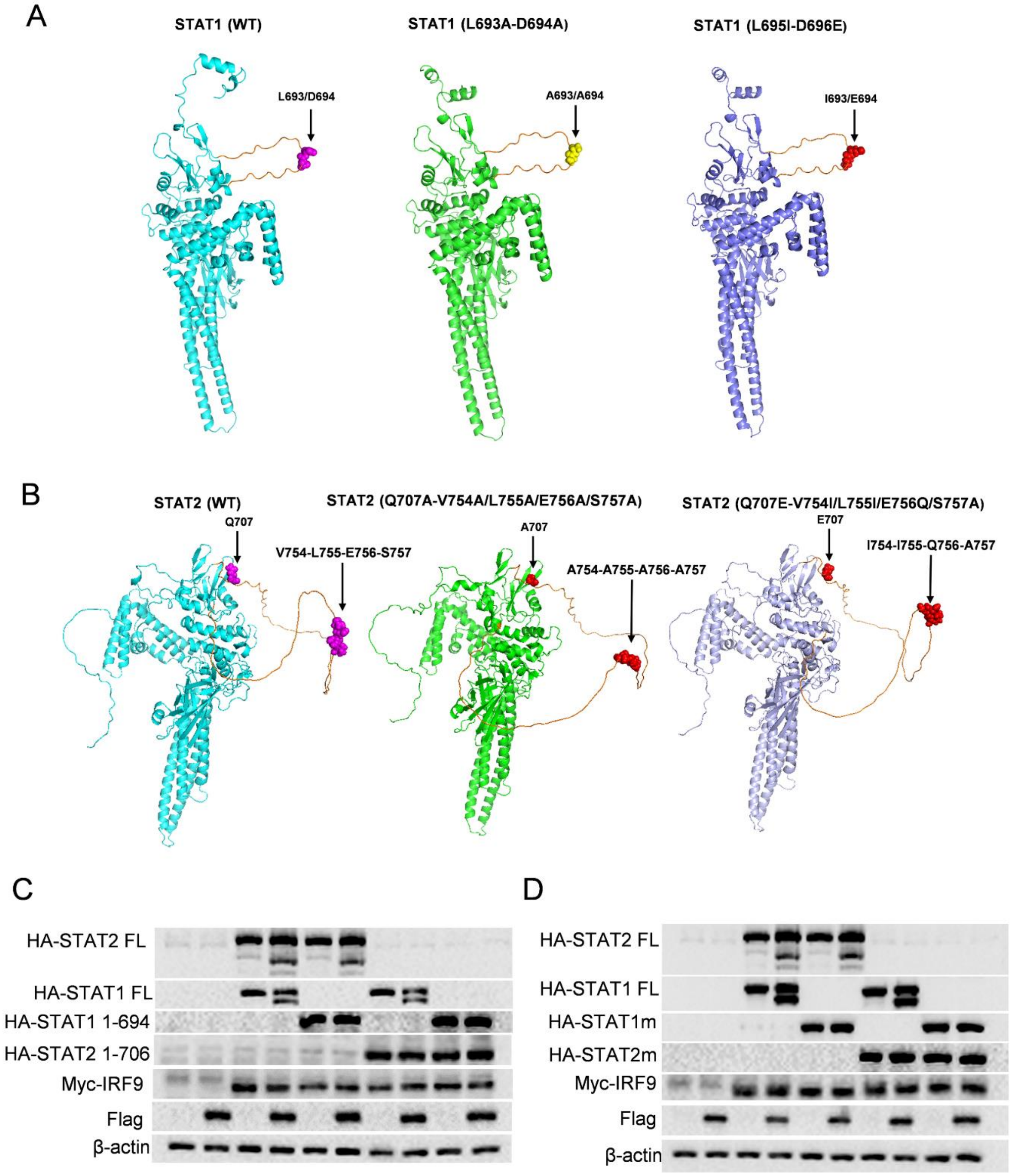
A
STAT1 (WT)
STAT1 (L693A-D694A)
STAT1 (L695I-D696E)
L693/D694
A693/A694
I693/E694
B
STAT2 (WT)
STAT2 (Q707A-V754A/L755A/E756A/S757A)
STAT2 (Q707E-V754I/L755I/E756Q/S757A)
Q707
V754-L755-E756-S757
A707
A754-A755-A756-A757
E707
I754-I755-Q756-A757
C
HA-STAT2 FL
HA-STAT1 FL
HA-STAT1 1-694
HA-STAT2 1-706
Myc-IRF9
Flag
β-actin
D
HA-STAT2 FL
HA-STAT1 FL
HA-STAT1m
HA-STAT2m
Myc-IRF9
Flag
β-actin
